# Supplementary material for: Non-small Cell Lung Cancer Epigenomes Exhibit Altered DNA Methylation in Smokers and Never-smokers
Source: Genomics Proteomics Bioinformatics. 2023 Sep 22;21(5):991–1013. doi: 10.1016/j.gpb.2023.03.006 (PMC10928376; doi:10.1016/j.gpb.2023.03.006)
Supplement: Supplementary File S1 — Supplementary information supporting “Non-small cell lung cancer epigenomes exhibit altered DNA methylation in smokers and never-smokers”. [file mmc1.docx]

**File S1 Supplementary information supporting “Non-small cell lung cancer epigenomes exhibit altered DNA methylation in smokers and never-smokers”**

## Genome-wide methylation

There was no significant difference in the mean genome-wide CpG methylation level or the proportion of CpGs lowly methylated or highly methylated (< 30% or > 70%) based on sample malignancy, even when an unusually lowly methylated normal sample, P2385_N_UC, was excluded (*P* > 0.05, Wilcox test) (Figure 1A, Figure S2A). However, there were still significantly more intermediately methylated CpGs in primary non-small cell lung cancer (NSCLC) than in normal lung without P2385_N_UC (Wilcox *P* < 0.001). There was no significant difference for any of the metrics between smokers and never-smokers within normal lung or tumor samples (excluding patients with unconfirmed smoking status) or based on sample malignancy within smoking status, except for intermediately methylated CpGs in smokers (Wilcox *P* < 0.05). Finally, there was no significant difference in any of the metrics among tumor samples based on tumor stage (Kruskal–Wallis test), patient sex (Wilcox test), or percentage tumor (Pearson correlation), nor was there any significant difference in the by-patient normal–tumor shift in any of the metrics based on clinicopathologic data, except percentage tumor.

There was no significant difference in the Pearson correlation between normal lung and tumor samples from the same patient based on tumor stage or subtype (Kruskal–Wallis test), smoking status (Wilcox test), or percentage tumor (Pearson correlation) when all patients were included.

There was not a significant difference between never-smokers and smokers in the average CpG methylation level over promoters, within either normal lung or primary NSCLC samples (Wilcox *P* > 0.5).

There was also no significant difference between never-smokers and smokers in either the number of filtered methylation sensitive restriction enzyme sequencing (MRE-seq) reads or the number of sampled cut sites within normal or tumor samples (Wilcox *P* > 0.05).

## Comparison of methylCRF to previously published results and MRE-seq/MeDIP-seq data

Sixteen samples profiled with methylCRF, including normal endometrium and five types of endometrial cancer, were downloaded [1] (Figure S3A and B). Although the average methylation level for the normal endometrium was higher than for the tumor samples, the difference could be slight. Furthermore, methylation was lowest in the laser-microdissected tumor components, which likely have higher purity.

Additionally, analysis of MRE-seq data independently confirmed that the primary NSCLC samples were more hypomethylated than normal lung. The number of sampled restriction enzyme cut sites was greater in tumor samples regardless of read depth, indicating that more cut sites were unmethylated in tumors (Wilcox *P* < 0.001 for sampled cut sites, *P* = 0.60 for number of reads) (Figure S3C).

In comparison to all possible MRE cut sites, CpGs exclusive to tumor samples (*n* = 1,162,595) were enriched in intergenic regions and over repeats, while CpGs exclusive to normal lung (*n* = 81,623) were enriched over genes (Figure S3D). Due to known limitations of methylCRF, the bias toward hypomethylated sites in these regions may lead to an underestimation of total genome-wide hypomethylation in primary NSCLC. As expected, both normal-exclusive and tumor-exclusive cut sites were less frequently represented than those that were shared (Figure S3E and F).

Finally, we performed principal component analysis (PCA) on methylated DNA immunoprecipitation sequencing (MeDIP-seq) and MRE-seq reads per kilobase per million mapped reads (RPKM) in 500 bp bins across the genome. MRE-seq much more clearly separated normal lung from tumor samples, with lower variability between normal samples (Figure S3G and H).

## Selection of DMR *Q* value threshold

The differentially methylated region (DMR) *Q* value threshold was selected from among four potential thresholds: 1E-2 (M&M default threshold), 1E-3, 1E-4, and 1E-5. Although the proportion of DMRs in each *Q* value interval was similar for patient-matched normal *vs.* tumor DMRs, normal *vs.* normal DMRs, and unpaired normal *vs.* tumor DMRs (Figure S6A), the number of DMRs was far smaller for normal *vs.* normal comparisons, particularly at higher *Q* values. (Figure S6B). The exception was Patient 4999, which had a similar number of DMRs as the normal *vs.* normal comparisons.

For all DMRs below each of the potential *Q* value thresholds, a false positive ratio was calculated for each patient by dividing the mean number of DMRs between the normal lung sample and other normal samples by the number of DMRs in comparison to the patient-matched tumor (Figure S6C). Patient 4999 was excluded from this analysis due to its unusually low number of DMRs (Figure S6D). For some samples, the false positive ratio increased as the threshold became more stringent, with a greater increase below 1E-3 than between 1E-2 and 1-E3. With a *Q* value threshold of 1E-3, the greatest by-patient false positive ratio was 16% (excluding Patient 4999).

Next, for all DMRs below each potential *Q* value threshold, we analyzed the change in mean CpG methylation level over the DMR between its normal lung and tumor sample as predicted using methylCRF. With a lower *Q* value threshold, DMRs had a greater change in methylation in the expected direction (Figure S6E and F). With a *Q* value threshold of 1-E3, 88% of hypomethylated DMRs and 86% of hypermethylated DMRs had a methylation change > 10% in the expected direction. Of the hypermethylated DMRs with a methylation change < 10%, 1036 were on chromosome 14 of Patient 9890, 739 were on chromosome 3 of Patient 8666, and 405 were on chromosome 8 of Patient 6356, which had unusually high DMR densities (Figure 5B).

Finally, we calculated the proportion of DMRs below each *Q* value threshold that were shared between patients (Figure S6G). As the threshold became more stringent, the proportion of DMRs that were shared decreased, but the drop was largest from 1E-2 to 1E-3.

## Number of DMRs per comparison

Neither the total number of DMRs nor the proportion hypomethylated correlated with any patient clinicopathologic data, including tumor purity (Pearson correlation), tumor stage or subtype (Kruskal–Wallis test), and smoking status (Wilcox test, *P* > 0.05). Pearson correlations with change in average CpG methylation level between patient-matched normal and tumor samples (directional or absolute) were also not significant (*P* > 0.05).

## Normal *vs.* normal DMRs

There were 12,160 DMR instances between normal samples (0.7% of all instances versus 21% of all comparisons) across 4626 unique DMRs. However, only 307 unique DMRs were exclusive to normal *vs.* normal comparisons (0.1%). 7114 of the normal *vs.* normal DMR instances were between P2385_N_UC and another normal sample (59% versus 20% of normal *vs.* normal comparisons), 97% of which were hypomethylated in the other sample compared to P2385_N_UC.

The normal *vs.* normal comparisons with P2385_N_UC had the highest number of total DMRs, and without them, the maximum number of normal *vs.* normal DMRs was 340.

Based on genome-wide methylation profiles, normal lung samples were more homogenous than primary NSCLC. Consistent with this observation, normal samples had a relatively uniform distribution of DMRs in comparison to all tumors and other normal lung samples, including the normal sample for the bronchioloalveolar carcinoma (Figure S7C). In contrast, the number of DMRs between each primary tumor and any normal sample varied considerably by tumor but was internally consistent (Figure S7D). This confirmed that some tumors were more epigenetically similar to normal lung, and that inter-tumor variability drove the variation observed in the number of patient-matched DMRs. In support of this observation, the number of DMRs between each tumor and its patient-matched normal sample strongly correlated with the number of DMRs between the tumor and other normal samples (Pearson correlation ≥ 0.91, *P* < 0.001, median and total number of hypomethylated and hypermethylated DMRs) and did not correlate with the number of DMRs between the normal and other tumor samples (*P* > 0.5 for all comparisons) (Figure S7E and F).

## Feature overlap

Many DMRs overlapped multiple genic features and were counted for both categories, either because 500 bp DMRs could span multiple features (as in the case of intergenic/exonic or intronic/exonic DMRs) or because feature definitions overlapped (*e.g.*, intergenic/promoter). 29% of intronic DMRs and 14% of intergenic DMRs also overlapped promoters, which was higher for hypermethylated than hypomethylated DMRs. In contrast, the overlap between intergenic and intronic regions was low.

## DMR density feature correlation

The density of genes and transcripts (all and protein-coding) over 1 Mb genome-wide windows was highly correlated, with a Pearson correlation > 85% for all pairwise comparisons. The correlation of each metric with DMR density over 1 Mb windows had a narrow range (< 4% for both hypomethylated and hypermethylated DMRs, *P* < 0.001), so the metric with the highest correlation (gene density) was used for downstream analyses.

The remaining window features were highly correlated (Figure S22A). The 18_Quies state was anticorrelated with all other ChromHMM states and gene, CpG, and repeat density. Active regulatory and transcribed ChromHMM states were highly correlated with each other and with gene, CpG, and repeat density, with the Polycomb states and the ZNF/Rpts and heterochromatin states forming two additional clusters.

We also performed PCA on the window features to determine which contributed the most to variation among windows. Principal component 1 (PC1) (45% of variance) was highly correlated with the quiescent state (Pearson correlation 0.84, *P* = 0) and anticorrelated with gene and CpG density (Pearson correlation −0.82 and −0.89, *P* = 0) and other active states (Figure S22B and C). PC2 (13% of variance) separated quiescent and active states from repressed states, while PC3 (7% of variance) separated the Polycomb-repressed states from the heterochromatin and ZNF/Rpts states and repeat density (Figure S22D and E).

## Repeats

In both directions, 96% of the repeats that overlapped DMRs overlapped only one unique DMR. The maximum was 9 unique DMRs per repeat (a GSATX satellite element, chr12:38545411–38551396).

## Reference

[1] Li J, Xing X, Li D, Zhang B, Mutch DG, Hagemann IS, et al. Whole-genome DNA methylation profiling identifies epigenetic signatures of uterine carcinosarcoma. Neoplasia 2017;19:100–11.
