# Supplementary figures and images for: Non-small Cell Lung Cancer Epigenomes Exhibit Altered DNA Methylation in Smokers and Never-smokers
Source: Genomics Proteomics Bioinformatics. 2023 Sep 22;21(5):991–1013. doi: 10.1016/j.gpb.2023.03.006 (PMC10928376; doi:10.1016/j.gpb.2023.03.006)

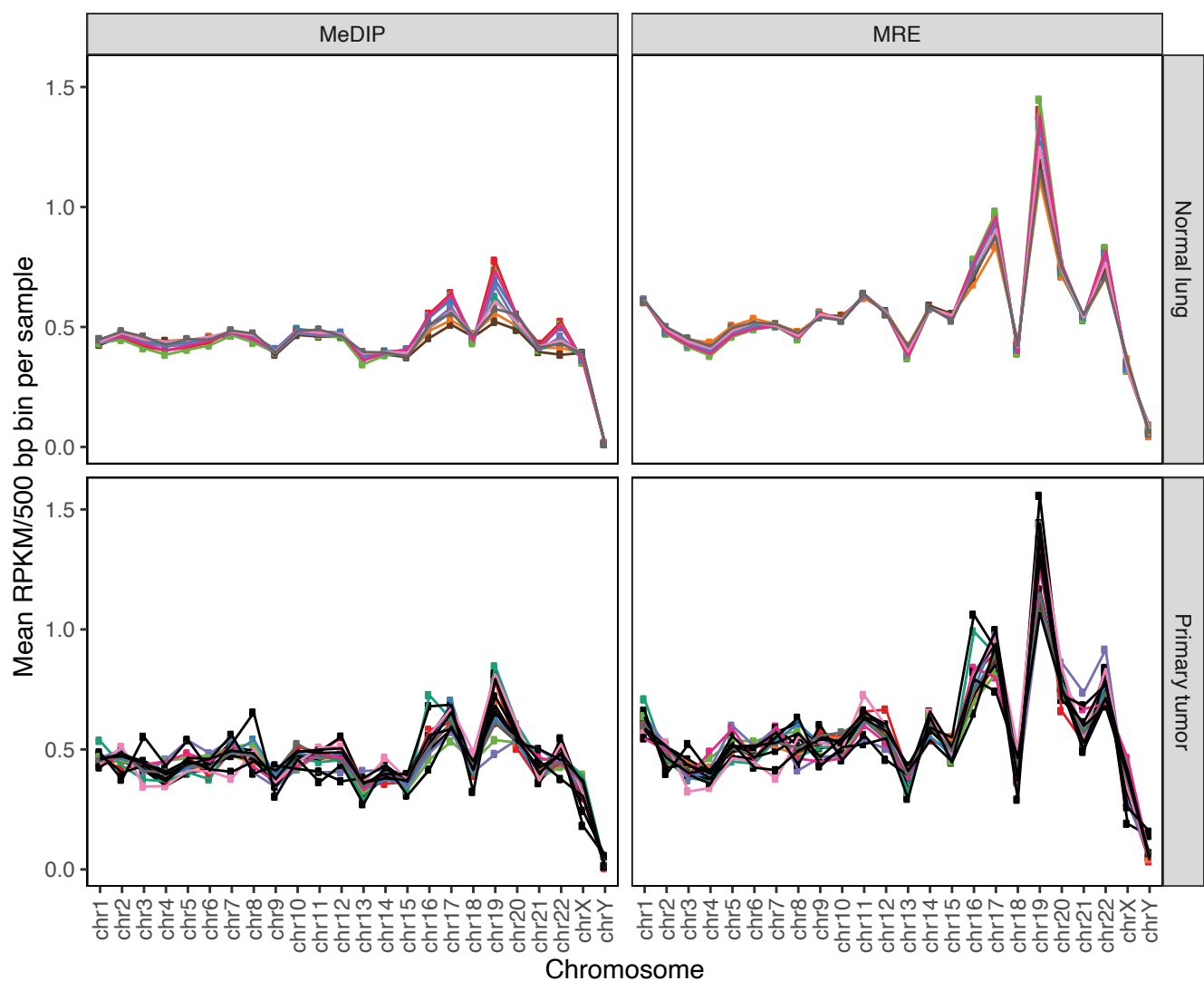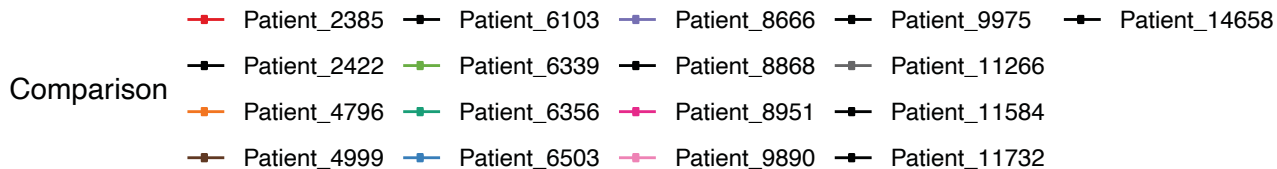

Supplement: Supplementary Figure S1 — Read coverage over chromosomes Mean MeDIP-seq and MRE-seq RPKM per 500 bp bin by sample for each chromosome, split by sample malignancy. MeDIP-seq, methylated DNA immunoprecipitation sequencing; MRE-seq, methylation sensitive restriction enzyme sequencing; chr, chromosome; RPKM, Reads Per Kilobase per Million mapped reads. [file mmc2.pdf]

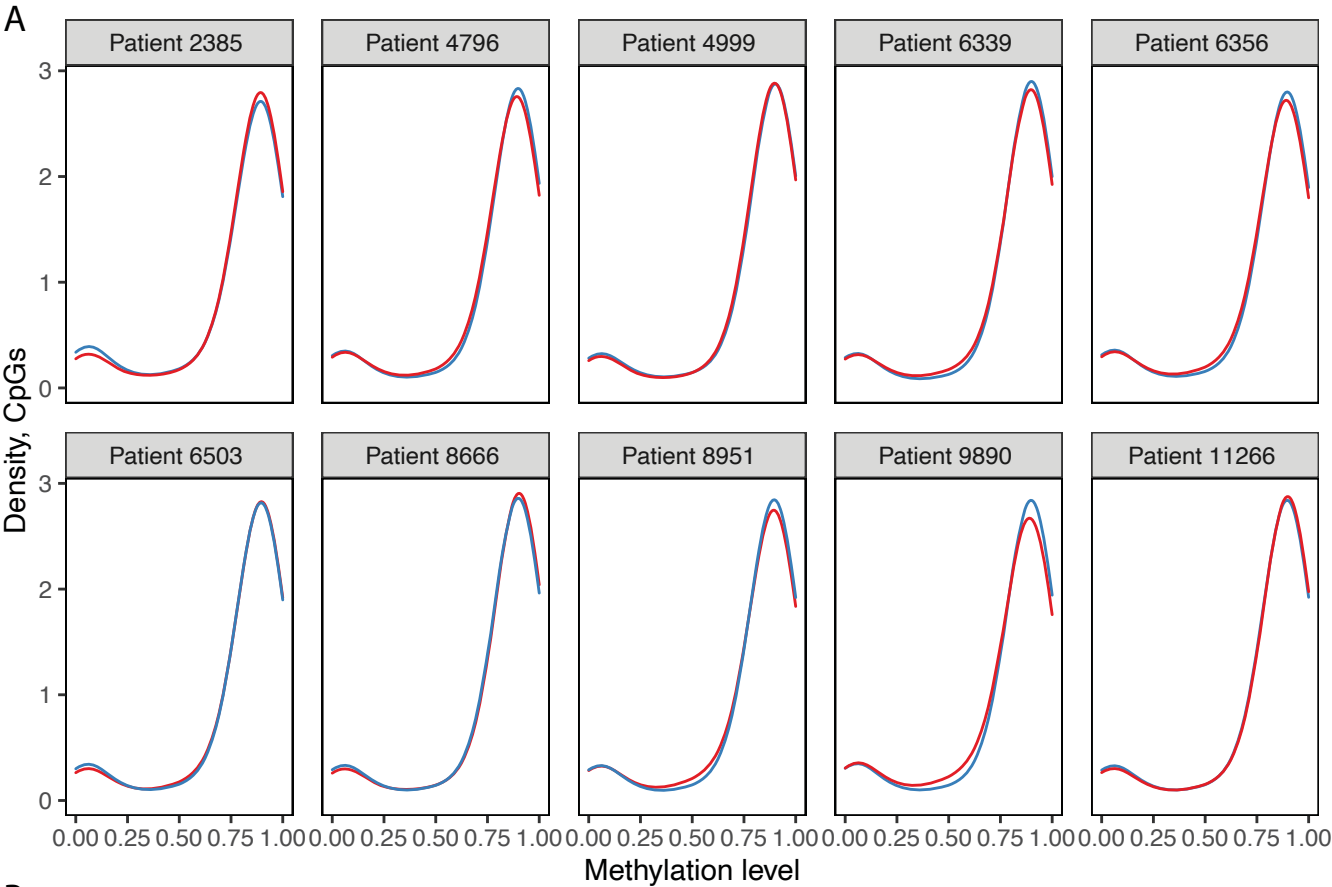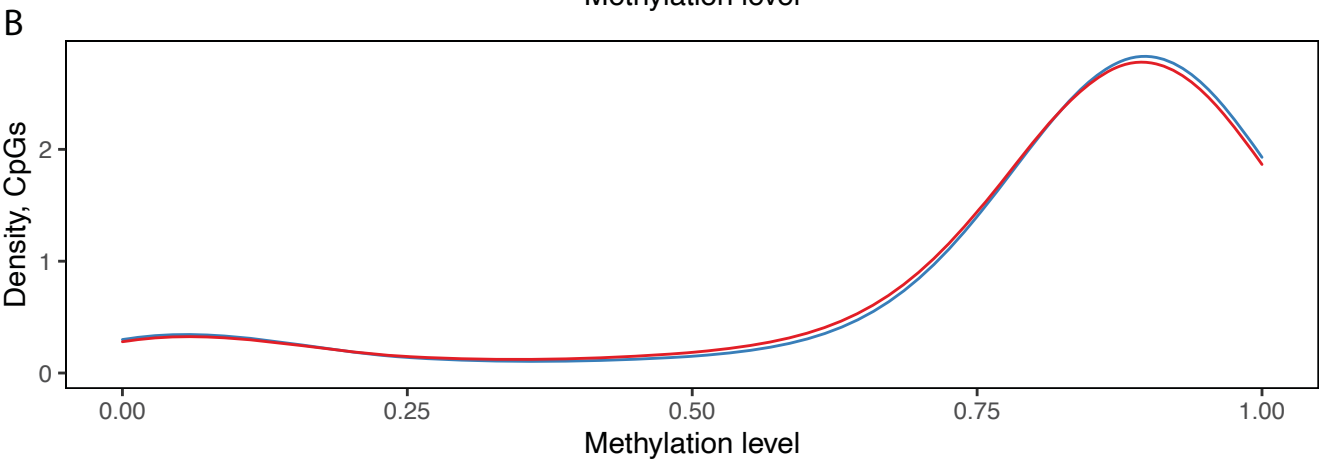

Supplement: Supplementary Figure S2 — CpG methylation level distribution by sample and patient, averaged across disease state A. Distribution of the number of CpGs at each methylation level in the normal lung and primary tumor samples for each patient. Samples are colored by malignancy, where blue is normal and red is tumor. B. Distribution of the number of CpGs at each methylation level as assigned by methylCRF, averaged over all samples by malignancy, where blue is normal and red is tumor. [file mmc3.pdf]

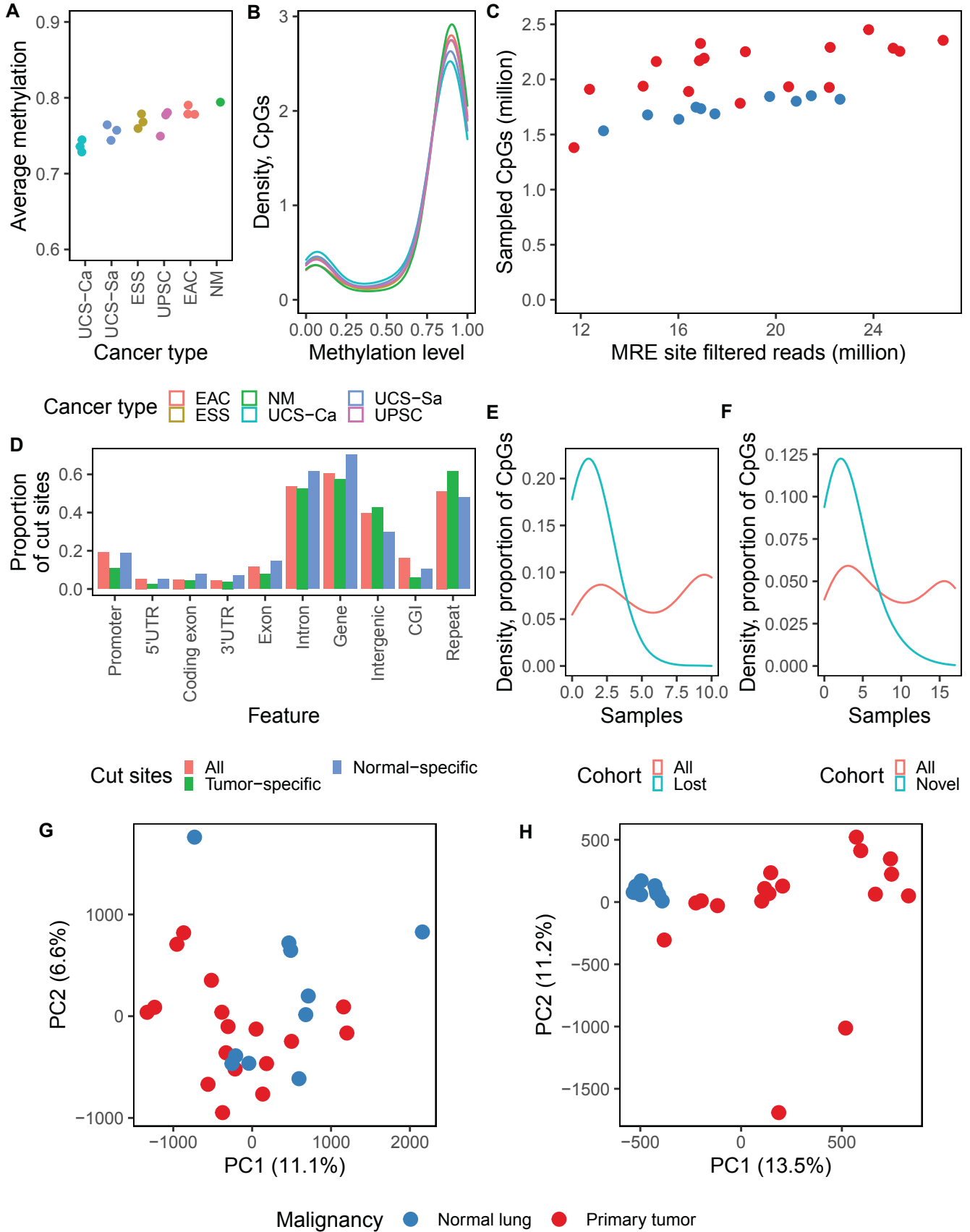

Supplement: Supplementary Figure S3 — DNA methylation alterations using MRE-seq and MeDIP-seq data and comparison to previously published samples A. Average genome-wide CpG methylation level per sample, ordered by mean methylation level per cancer type. B. Distribution of the number of CpGs at each methylation level as assigned by methylCRF, averaged over all samples by cancer type. Both A and B samples from [63], including NM, UCS-Ca, UCS-Sa, EAC, ESS, and UPSC. NM, 10 pooled normal endometrium samples; UCS-Ca, uterine carcinosarcoma carcinoma; UCS-Sa, uterine carcinosarcoma sarcoma; EAC, endometrioid carcinoma; ESS, endometrial stromal sarcoma;UPSC, endometrial serous carcinoma. C. Number of sampled MRE CpG cut sites versus the number of MRE-seq reads mapped to cut sites per sample, colored by sample malignancy (see legend below Figure S3G and H, Wilcox P when comparing normal vs. tumor sampled sites: P < 0.001). D. Location of MRE CpG cut sites, where “All” is all possible MRE cut sites. E. Distribution of the proportion of MRE CpG cut sites sampled in each number of normal samples, where “All” is all MRE cut sites sampled in normal samples and “Lost” is MRE cut sites sampled only in normal samples. F. Distribution of the proportion of MRE CpG cut sites sampled in each number of tumors, where “All” is all MRE cut sites sampled in tumors and “Novel” is MRE cut sites sampled only in tumors. G. PCA on MeDIP-seq RPKM per 500 bp bin, colored by sample malignancy. H. PCA on MRE-seq RPKM per 500 bp bin, colored by sample malignancy. [file mmc4.pdf]

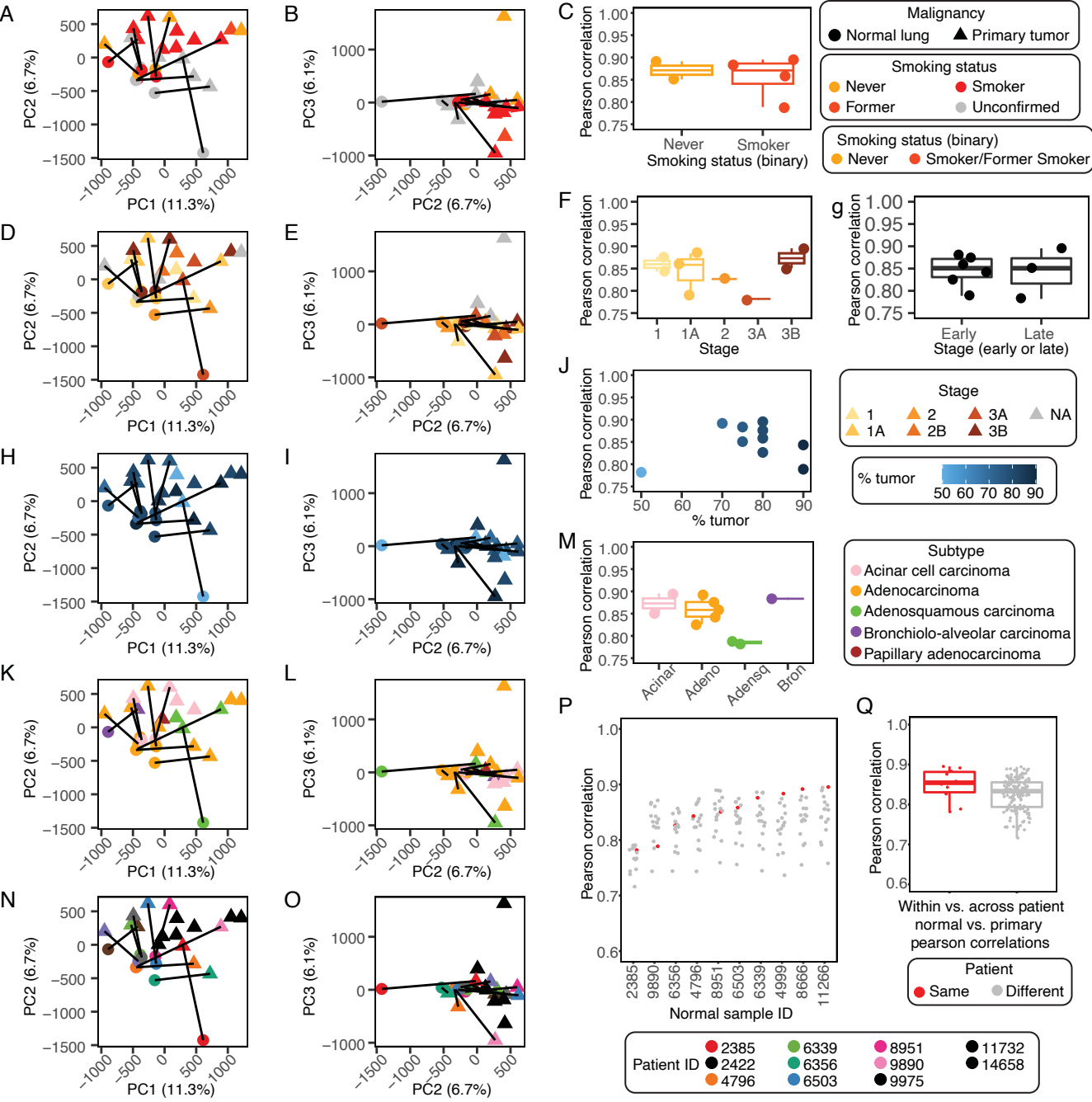

Supplement: Supplementary Figure S4 — Global patient methylation changes by patient clinicopathologic data PCA on normal lung and primary tumor samples, using the mean methylation over genome-wide 1 kb windows as features. Sample malignancy is indicated by shape (legend at top right). Axis titles display the amount of variance explained by each PC. Normal and primary tumor samples from the same patient are connected with a line. PCA plots are colored by (A and B) smoking status (legend to the right of panel C), (D and E) tumor stage (legend to the right of panel J), (H and I) percent tumor (legend to the right of panel J), (K and L) subtype (legend to the right of panel M), or (N and O) patient ID (legend below panel P). P2385_N_UC is the outlier on PC2, and P14658_T_NS is the outlier for PC3. Pearson correlation between paired normal and primary tumors, separated by (C) smoking status (see binary legend, Wilcox P = 1), (F) tumor stage, (G) binary tumor stage (early: stages 1, 1A, 2; late: stages 3A, 3B) (Wilcox P = 1), (J) percent tumor purity (Pearson product moment correlation P = 0.6079), or (M) subtype (Wilcox test comparing adenocarcinomas to adenosquamous carcinomas P = 0.09524). P. Pearson correlation between each normal lung sample and all tumors over genome-wide 1 kb windows, colored by whether the tumor was from the same patient and ordered by increasing correlation with the tumor from the same patient. Q. Pearson correlation between each normal lung sample and all tumors over genome-wide 1 kb windows, grouped according to paired or unpaired comparisons (Wilcox P = 0.06136). Acinar, acinar cell carcinoma; Adeno, adenocarcinoma; Adensq, adenosquamous carcinoma; Bron, bronchiolo-alveolar carcinoma; Unc., Unconfirmed. [file mmc5.pdf]

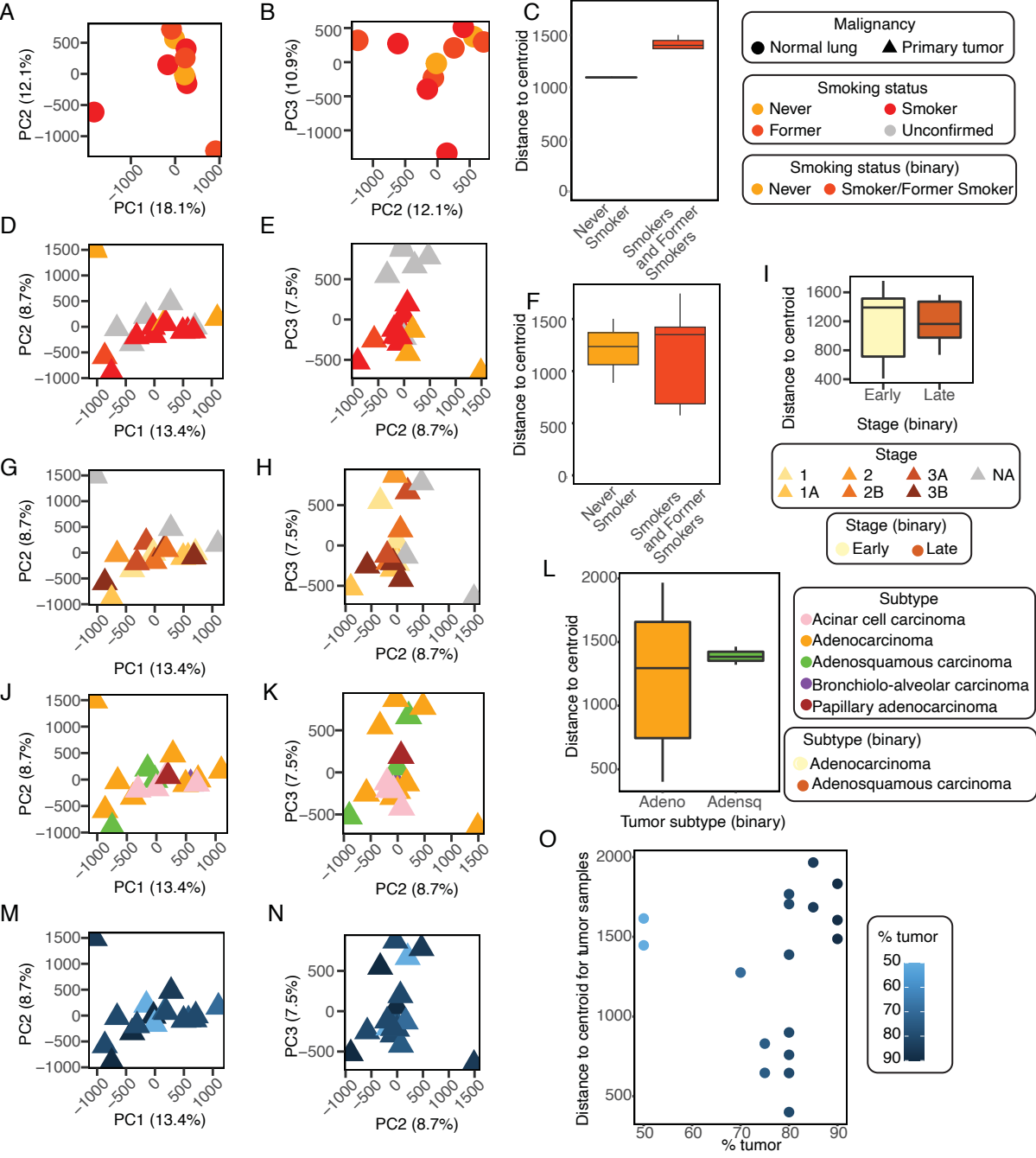

Supplement: Supplementary Figure S5 — CpG methylation variation by patient clinicopathologic data A and B. PCA on normal lung samples, using the mean methylation over genome-wide 1 kb windows as features, colored by smoking status (legend to the right of panel C). The outliers along PC1 are P2385_N_UC (left) and P4999_N_S (right), and the outlier along PC3 is P6356_N_UC. C. Distance to centroid for normal samples from smokers and from never-smokers (Wilcox P = 0.1002). D, E, G, H, J, K, M, N. PCA on tumor samples, using the mean methylation over genome-wide 1 kb windows as features, colored by (D and E) smoking status, (G and H) tumor stage (legend below panel I), (J and K) subtype (legend to the right of panel L), or (M and N) percent tumor purity (legend to the right of panel O). F. Distance to centroid of tumor samples from smokers and never-smokers (Wilcox P = 0.8636). I. Distance to centroid of early stage (1, 1A, 2, 2B) and late stage (3A, 3B) tumors (Wilcox P = 0.8981) L. Distance to centroid of Adeno and Adensq tumors (Wilcox P = 0.7676). O. Distance to centroid of tumor samples compared to percent tumor purity (Pearson product moment correlation P = 0.7664). [file mmc6.pdf]

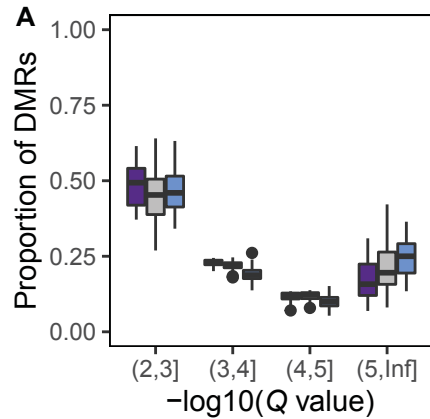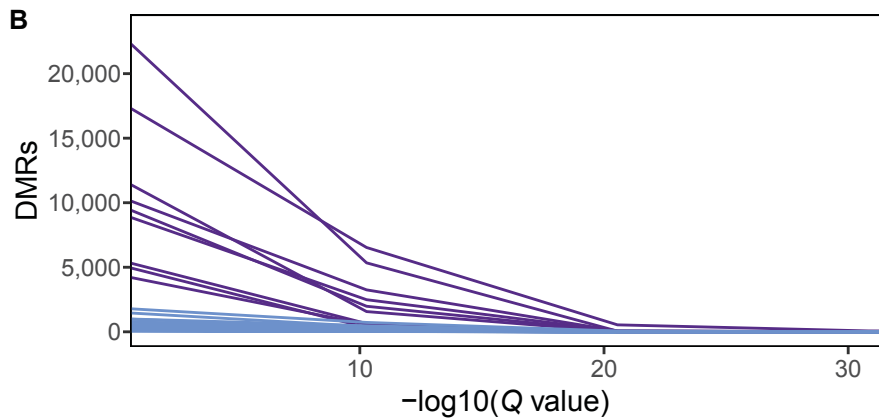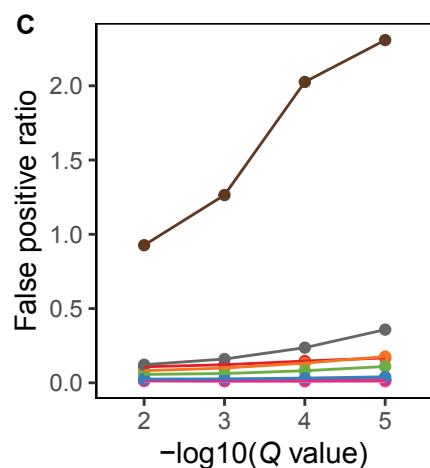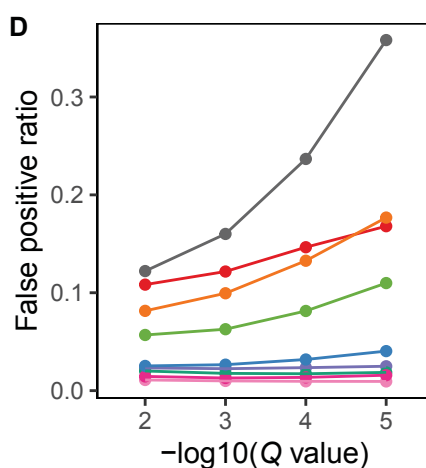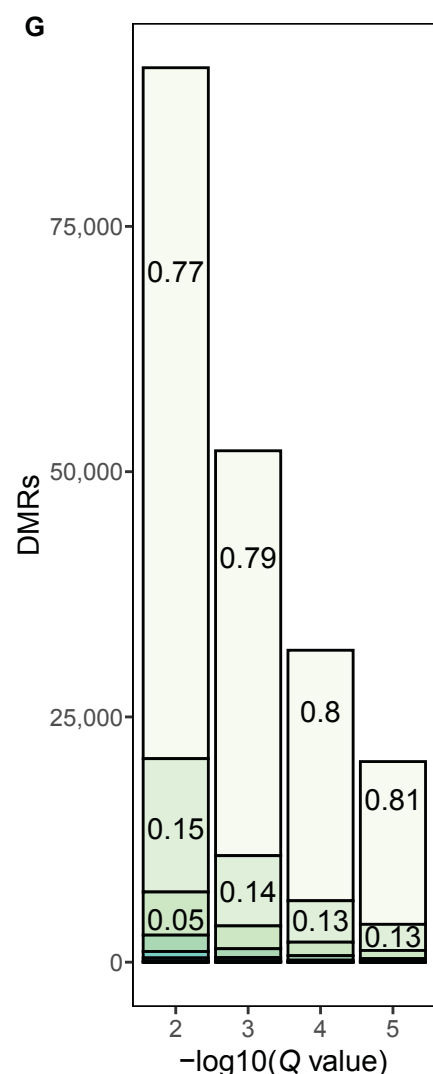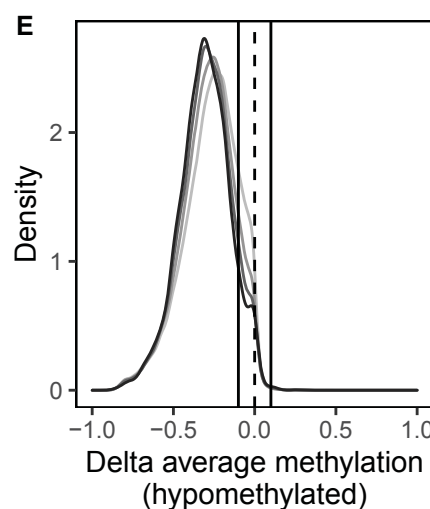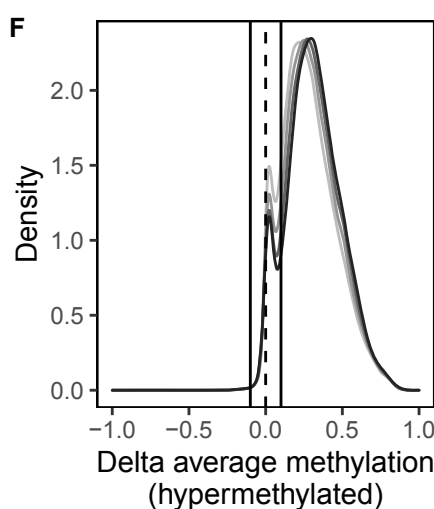

$-\log_{10}(\text{Q value})$  2 3 4 5

Patients 1 2 3 4 5 6 7 8 9

Supplement: Supplementary Figure S6 — Selection of DMR Q value threshold A. Proportion of DMRs within each Q value interval per comparison, by comparison set. B. Distribution of the number of DMRs by Q value for each comparison, colored by comparison set, with patient-matched normal vs. tumor and normal vs. normal lung comparisons only. A pseudocount of 1E-300 was added to the Q value to avoid undefined values, and DMRs with Q value < 1E-30 are not shown (n = 1433). C. and D. False positive ratio (mean number of DMRs in comparison to other normal samples divided by the number of DMRs in comparison to patient-matched tumor) for each normal sample for DMRs below each Q value threshold, for all samples (C) or excluding Patient 4999 (D). E. and F. Distribution of the change in mean CpG methylation level (as assigned by methylCRF) over each DMR between the patient-matched normal and tumor samples in which the DMR was identified, for DMRs below four Q value thresholds. Dashed line indicates no change in methylation; solid lines indicate a methylation change of 10%. G. Proportion of DMRs below each Q value threshold that are shared by each number of patients. Proportions < 0.05 are not shown. [file mmc7.pdf]

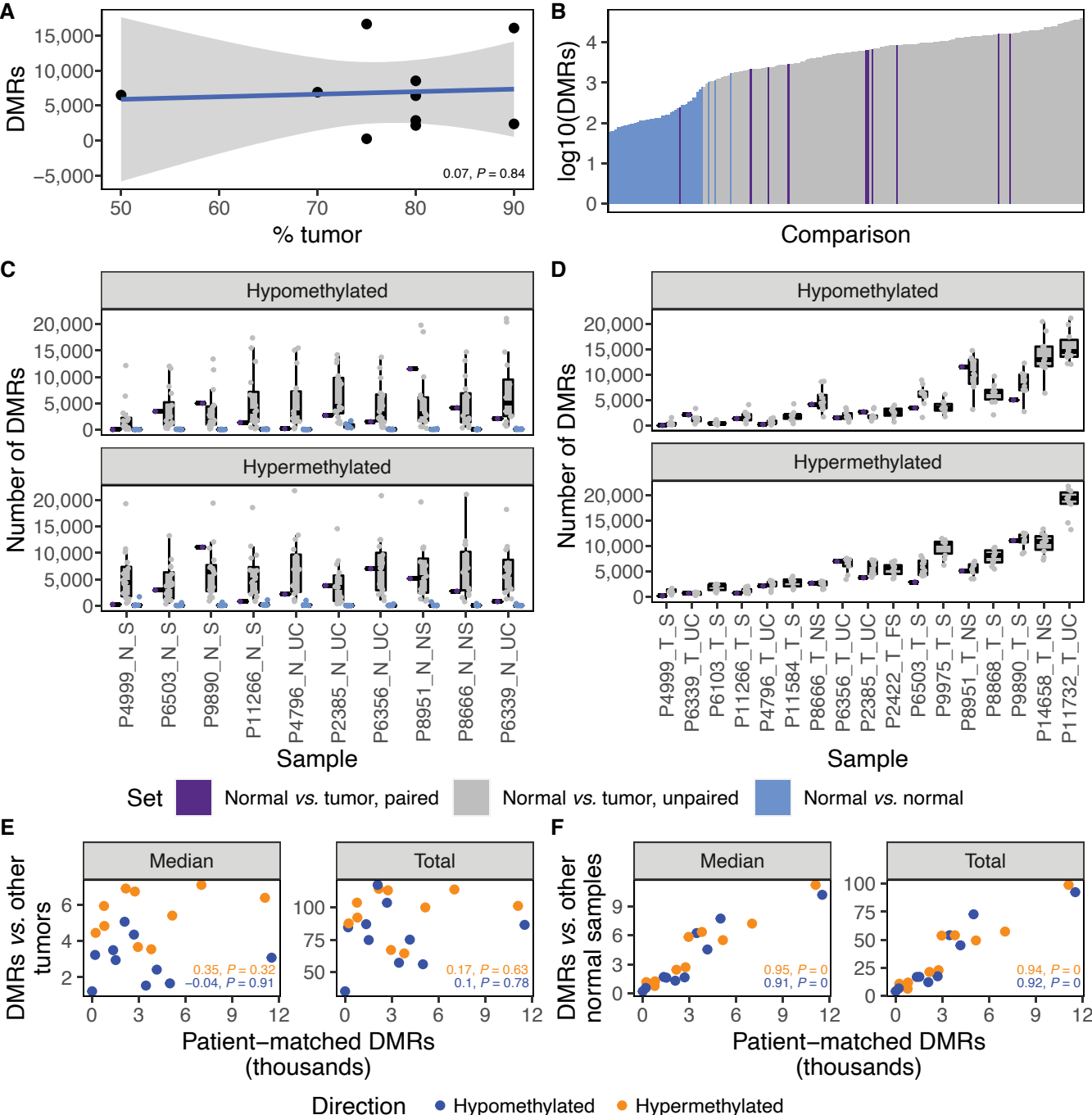

Supplement: Supplementary Figure S7 — Number of DMRs per comparison by normal and tumor sample A. Number of patient DMRs as a function of percent tumor. The linear relationship with shaded 95% confidence intervals is plotted. B. Number of DMRs per comparison (X-axis), colored by comparison set and ordered by increasing number of DMRs. C. and D. Number of DMRs identified between each normal sample and all other samples (C) and each tumor sample and all other samples (D), colored by comparison set, split by DMR direction. Samples are ordered by median number of DMRs across all comparisons. Normal vs. normal DMRs are counted for each normal sample. E. and F. Number of DMRs between patient-matched normal and tumor samples (X-axis) versus the number of DMRs between the normal sample and other tumors (E) and the tumor sample and other normal samples (F). Both the median and total number of DMRs in comparison to other samples are presented, and DMRs are split by direction. The Pearson correlation and P by DMR direction are listed on each graph. [file mmc8.pdf]

Number of DMRs

Never

Smoker

Smoking category

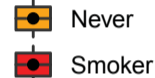

250  
200  
150  
100

P8666\_N\_NS

P8951\_N\_NS

P9890\_N\_S

P4999\_N\_S

P6503\_N\_S

P11266\_N\_S

Normal sample

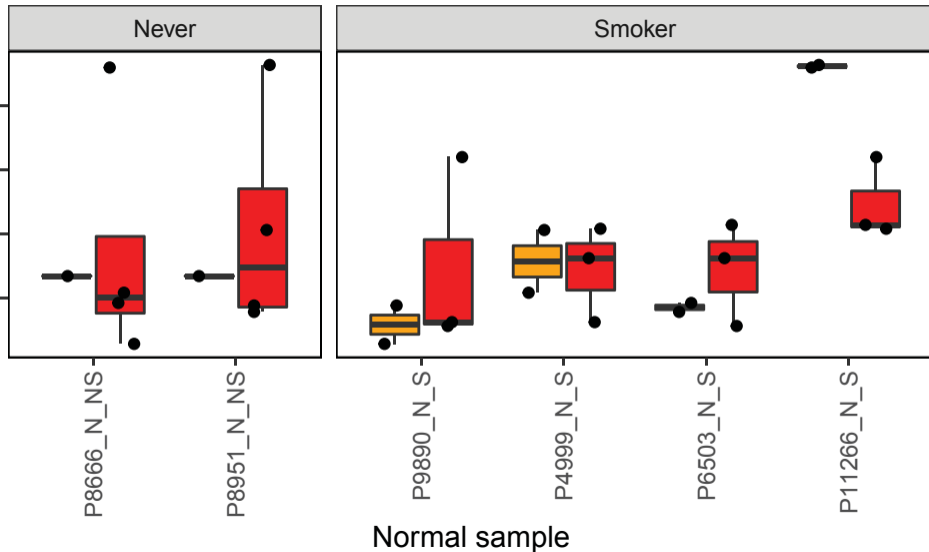

Supplement: Supplementary Figure S8 — Field effects of smoking Number of DMRs between normal lung samples. Normal samples are split into never-smokers and smokers by facets, and boxplot color indicates the smoking status of the second normal sample in the pair. Wilcox P > 0.1 for each normal sample. [file mmc9.pdf]

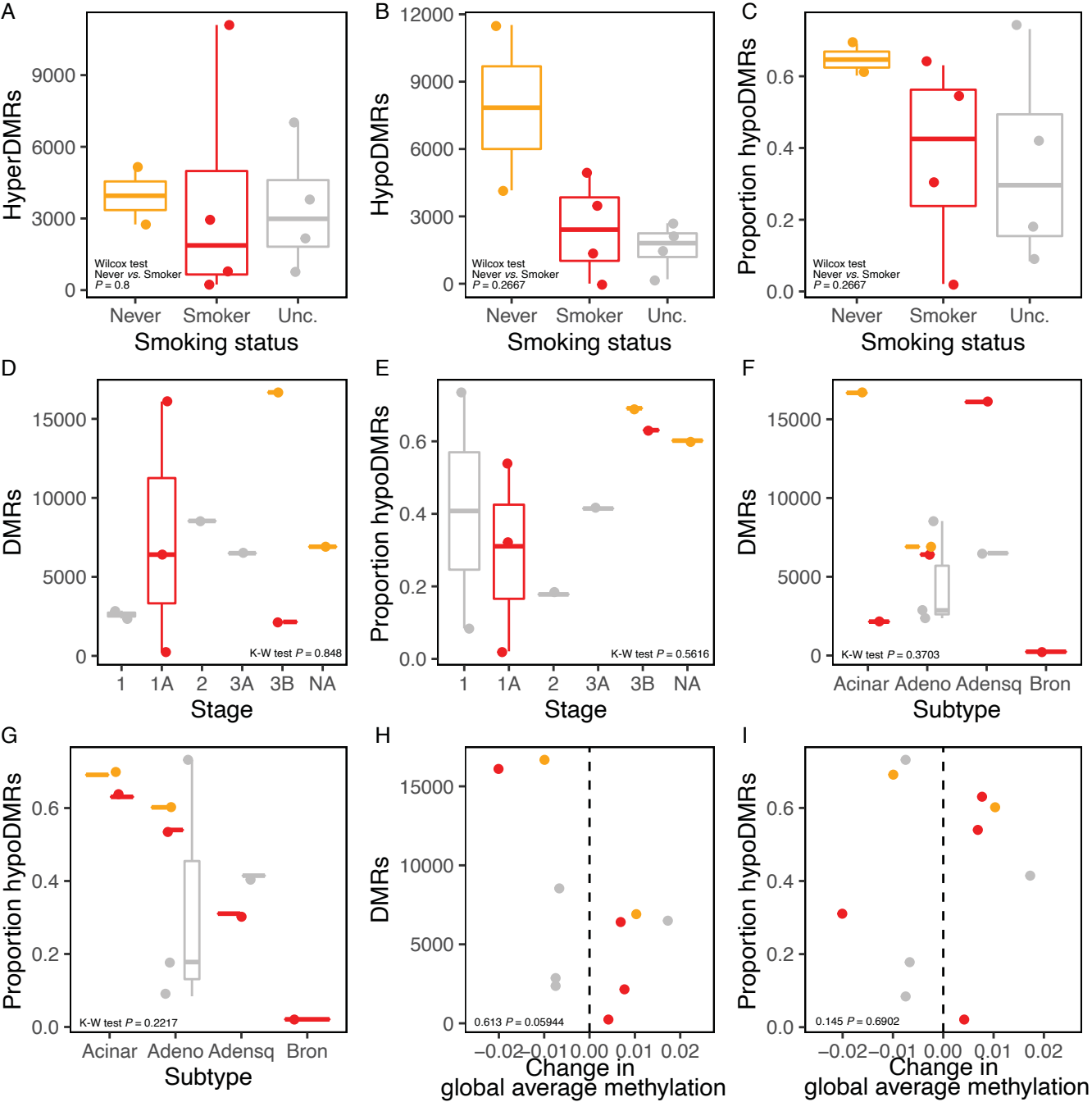

Smoking Status

Smoker/Former Smoker

Never Smoker

Unconfirmed

Supplement: Supplementary Figure S9 — Number of DMRs and proportion hypomethylated by patient clinicopathologic data Comparison of the number of (A) hyperDMRs and (B) and hypoDMRs for each patient according to smoking status. Wilcox tests comparing never-smokers (n = 2) to smokers (n = 4) were not significant for hyperDMRs (P = 0.8) or hypoDMRs (P = 0.2667). C. Comparison of the proportion of hypoDMRs by smoking status. Wilcox test comparing never-smokers (n = 2) to smokers (n = 4) was not significant (P = 0.2667). D. Comparison of the number of DMRs across patients according to tumor stage (n = 2, 3, 1, 1, 2, 1), colored by smoking status. Kruskal–Wallis P = 0.848. E. Comparison of the proportion of hypoDMRs across patients according to tumor stage (n = 2, 3, 1, 1, 2, 1), colored by smoking status. Kruskal–Wallis P = 0.5616. F. Comparison of the number of DMRs across patients according to tumor subtype (n = 2, 5, 2, 1) colored by smoking status. Kruskal–Wallis P = 0.3703. G. Comparison of the proportion of hypoDMRs across patients according to tumor subtype (n = 2, 5, 2, 1), colored by smoking status. Kruskal–Wallis P = 0.2217. H. Comparison of the number of DMRs across patients according to the change in genome-wide average CpG methylation level between normal lung and tumor sample, colored by smoking status. Dashed line indicates no change in methylation. Pearson’s product-moment correlation between number of DMRs and absolute change in methylation: 0.613, P = 0.05944. I. Comparison of the proportion of hypoDMRs across patients according to the change in genome-wide average CpG methylation level between normal lung and tumor sample, colored by smoking status. Pearson’s product-moment correlation between proportion of hypoDMRs and change in methylation: 0.145, P = 0.6902. [file mmc10.pdf]

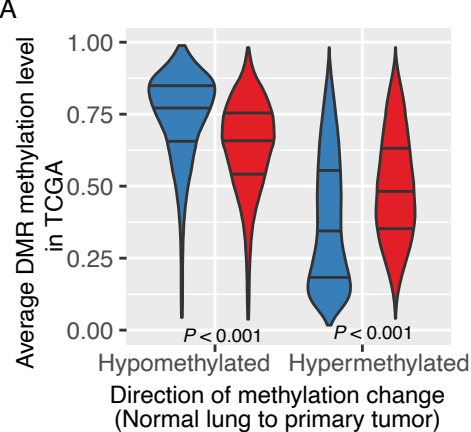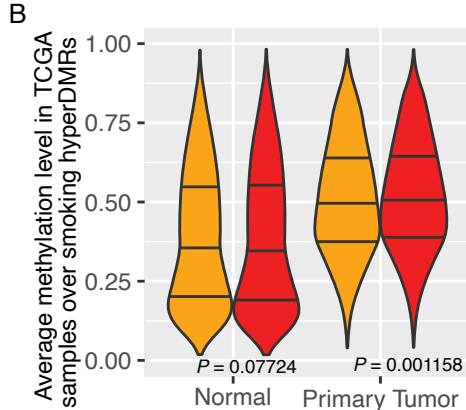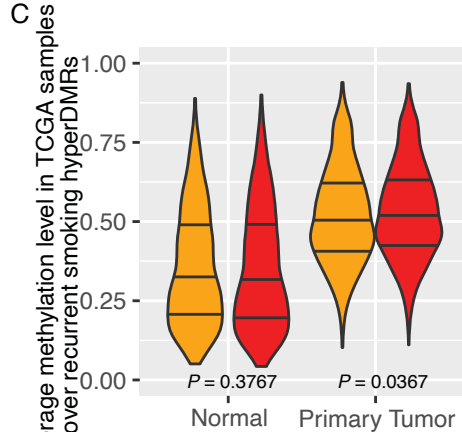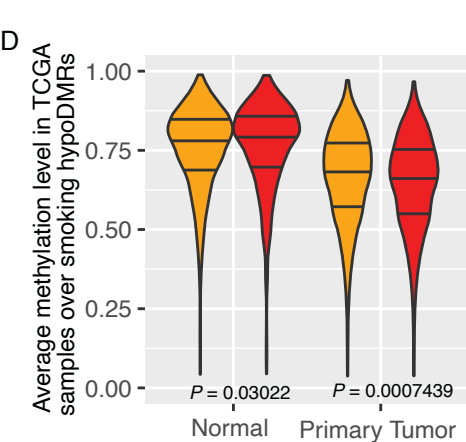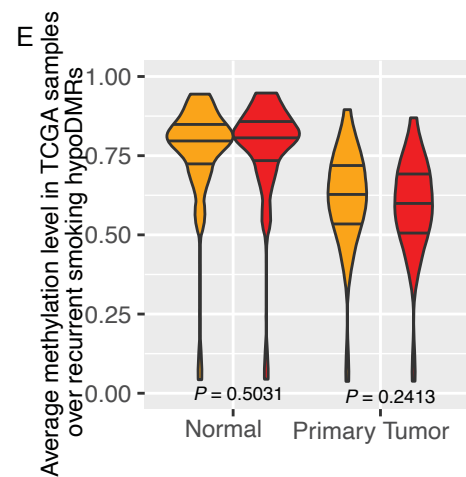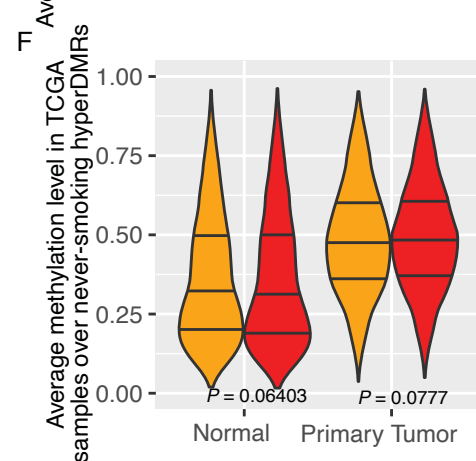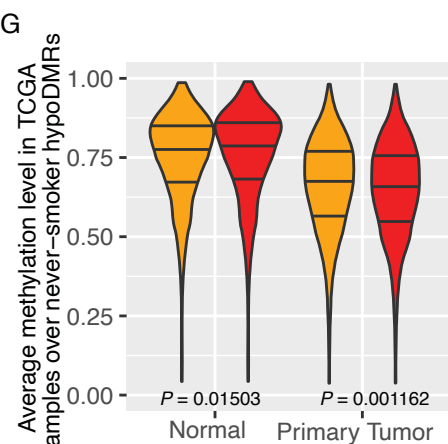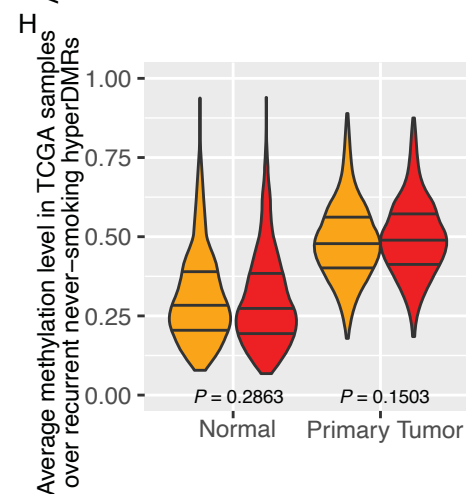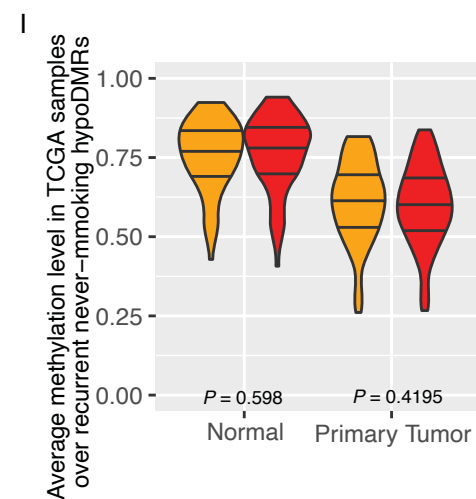

Sample Type ■ Normal ■ Primary Tumor

Smoking Status ■ Smoker/Former Smoker ■ Never Smoker/No Data

Supplement: Supplementary Figure S10 — DMR methylation status across TCGA LUAD samples A. Mean methylation level of each hypoDMR or hyperDMR across all TCGA LUAD samples, split by sample type. Violin plot lines indicate quartiles. Wilcox P < 0.001 between LUAD normal lung (blue) and primary tumor (red) for both hypoDMRs and hyperDMRs. Average methylation in TCGA samples, separated by sample type and smoking status, over (B) hyperDMRs identified in smoking patients from our cohort (n = 7410), (C) hyperDMRs identified in at least two smoking patients from our cohort (n = 967), (D) hypoDMRs identified in smoking patients from our cohort (n = 1137), (E) hypoDMRs identified in at least two smoking patients from our cohort (n = 55), (F) hyperDMRs identified in never-smoker patients from our cohort (n = 4063), (G) hypoDMRs identified in never-smoker patients from our cohort (n = 2014), (H) hyperDMRs identified in both never-smoker patients from our cohort (n = 501), or (I) hypoDMRs identified in both never-smoker patients from our cohort (n = 74). P from Wilcox tests comparing TCGA normal samples from smokers and never-smokers and comparing TCGA primary tumor samples from smokers and never-smokers are indicated. [file mmc11.pdf]

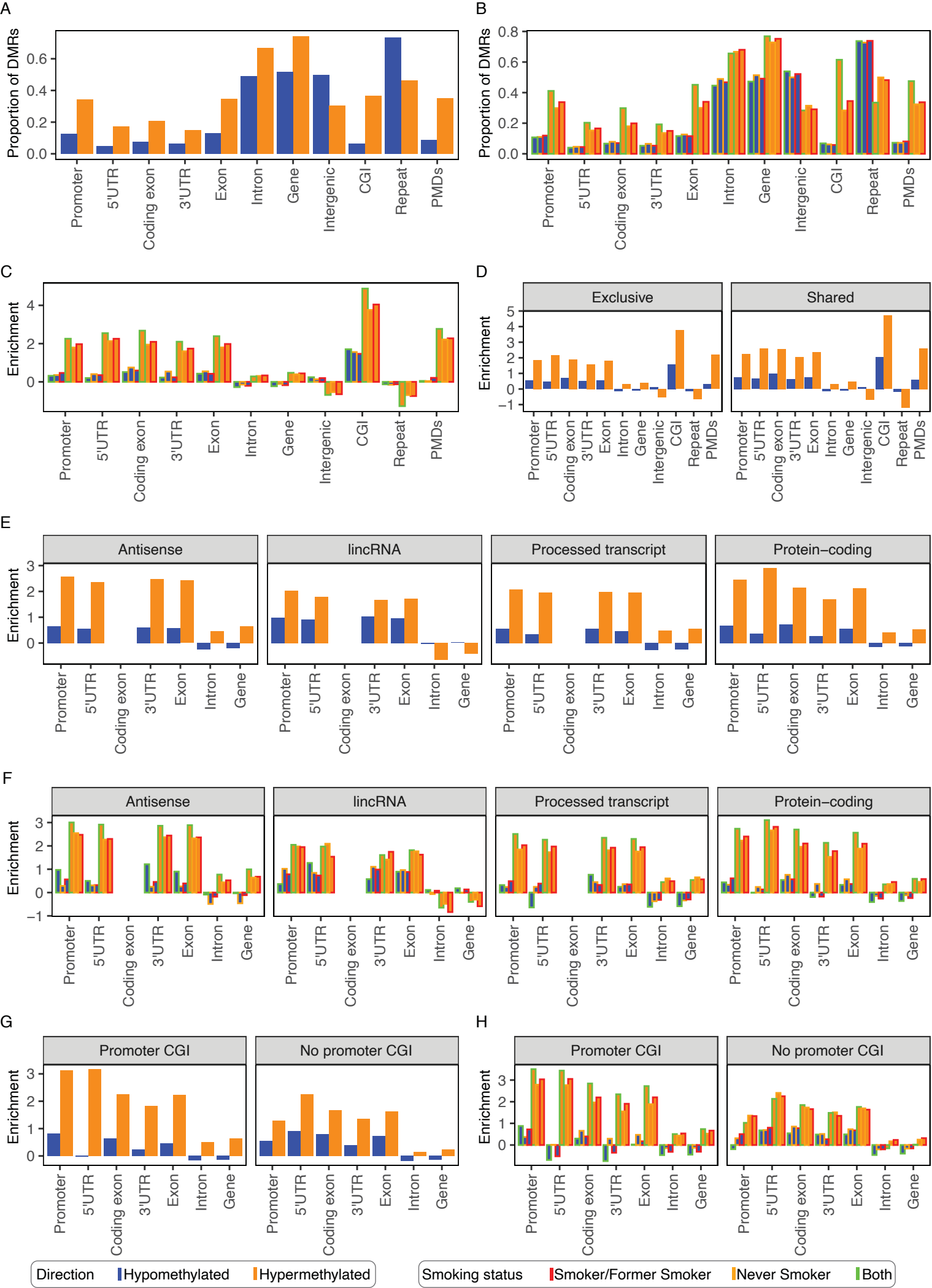

Supplement: Supplementary Figure S11 — Genomic location of DMRs by frequency and transcript information Proportion of patient-matched DMRs over genic features, intergenic regions, CGIs, and repeats, by (A) DMR direction, and (B) smoking status. C. Enrichment of patient-matched DMRs over genic features, intergenic regions, CGIs, and repeats, by DMR direction and smoking status. Log odds ratio enrichment of patient-matched DMRs over genic features, intergenic regions, CGIs, and repeats compared to the background distribution of 500 bp bins containing CpGs, by DMR direction (D, E, G) and smoking status (F, H). D. DMRs exclusive to a single patient or shared between multiple patients. Genic features restricted to four major categories of transcript biotype: protein-coding (n = 85,756, 45% of transcripts), processed transcript (n = 29,941, 16%), lincRNA (n = 11,773, 6%), and antisense (n = 9713, 5%). Protein-coding genic features split by whether the transcript promoter overlapped a CGI (yes: n = 52,222 transcripts, 64%; no: n = 29,357 transcripts, 36%). [file mmc12.pdf]

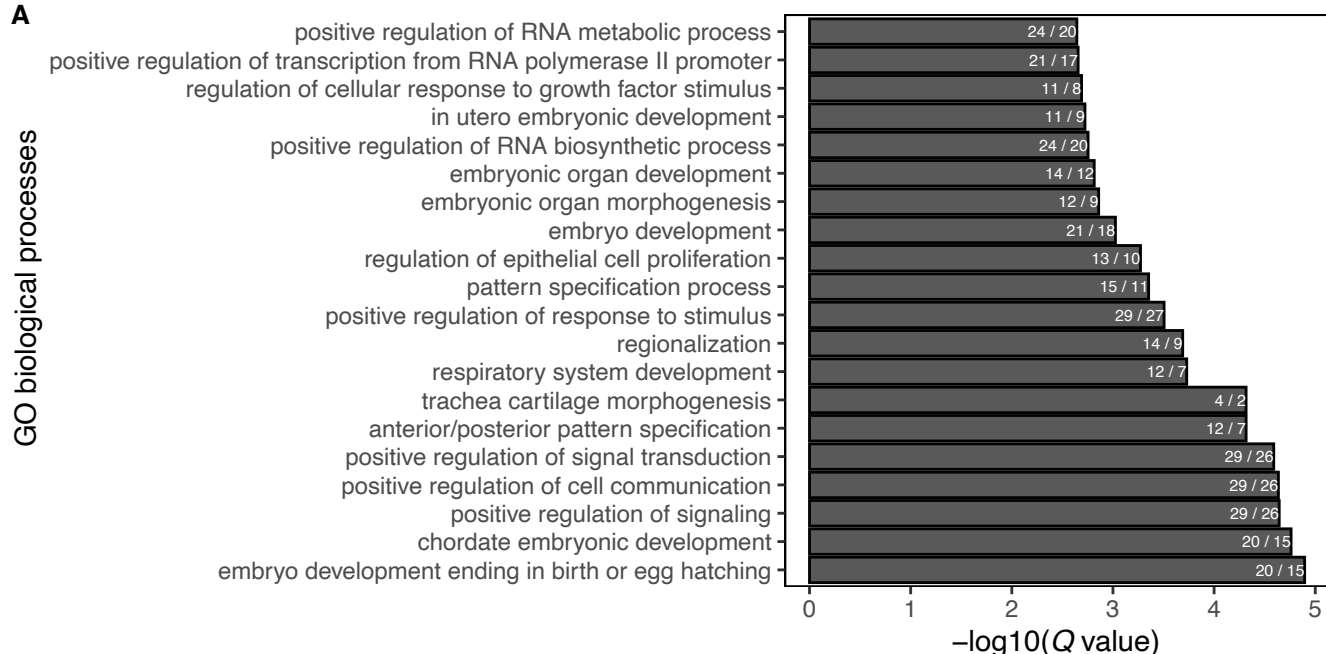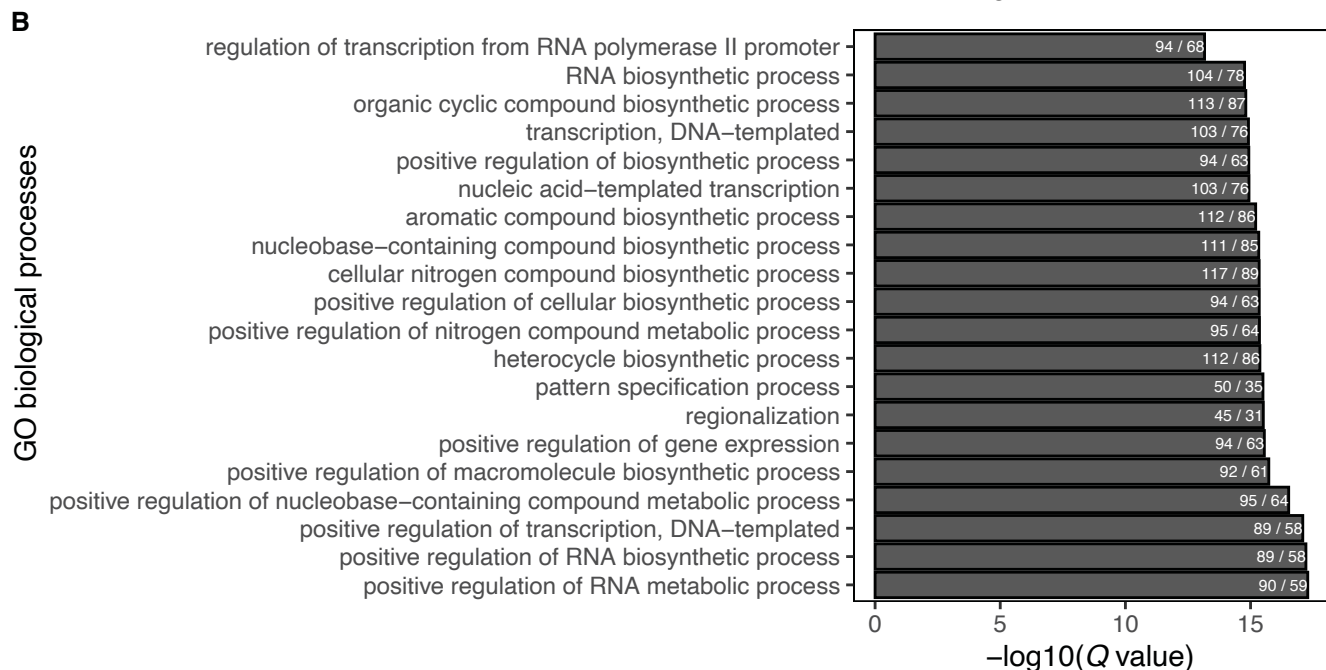

Supplement: Supplementary Figure S12 — Smoking status-specific DMR enrichment Top 20 significant GO biological processes as identified by GREAT (see Materials and methods) for hyperDMRs identified in at least 3 smokers and not in either never-smoker (of 40 terms) (A), hyperDMRs identified in both never-smokers and not in any smoker (of 200 terms) (B). Terms are ordered by FDR-corrected binomial Q and are labeled by the number of DMRs over the number of genes involved. [file mmc13.pdf]

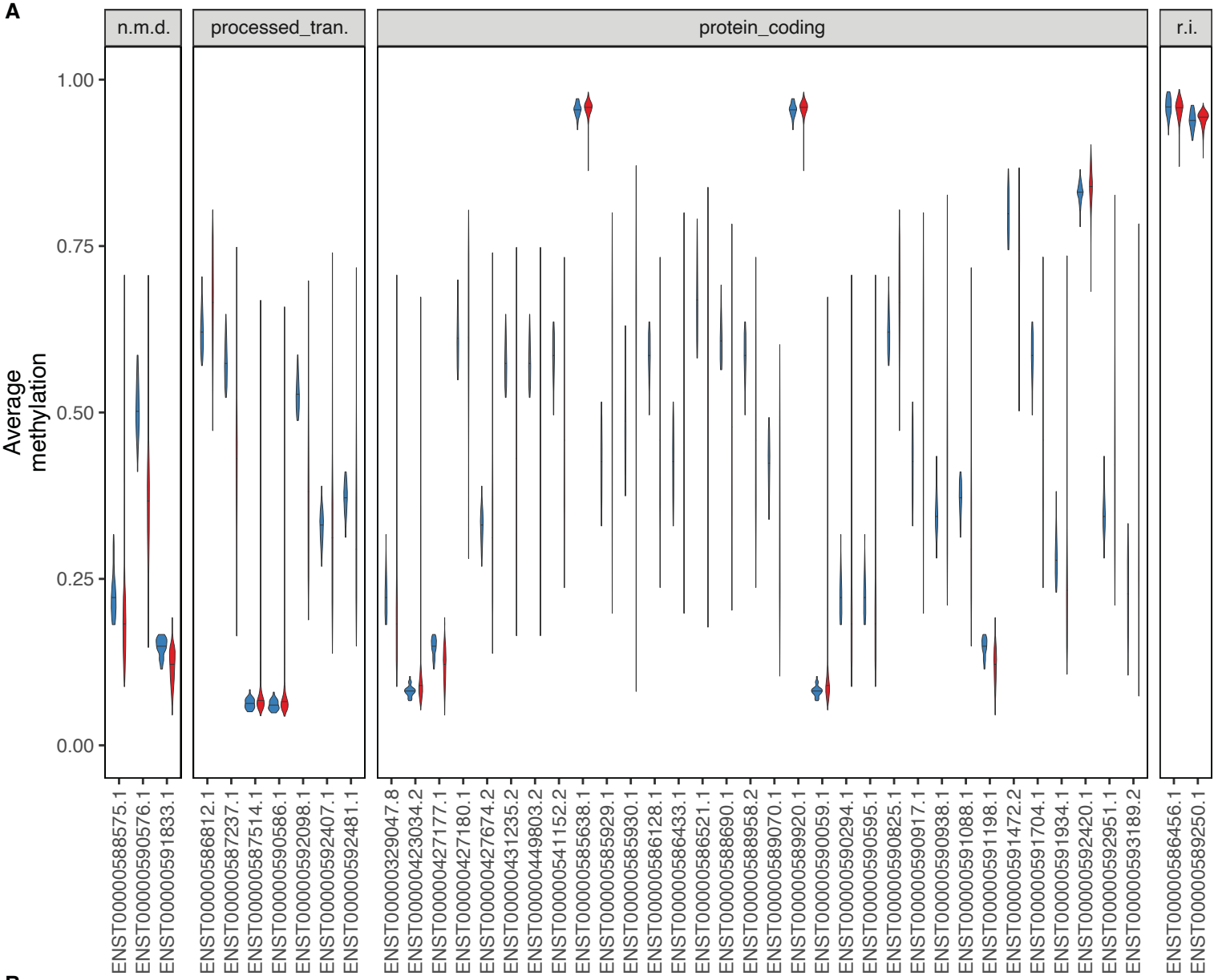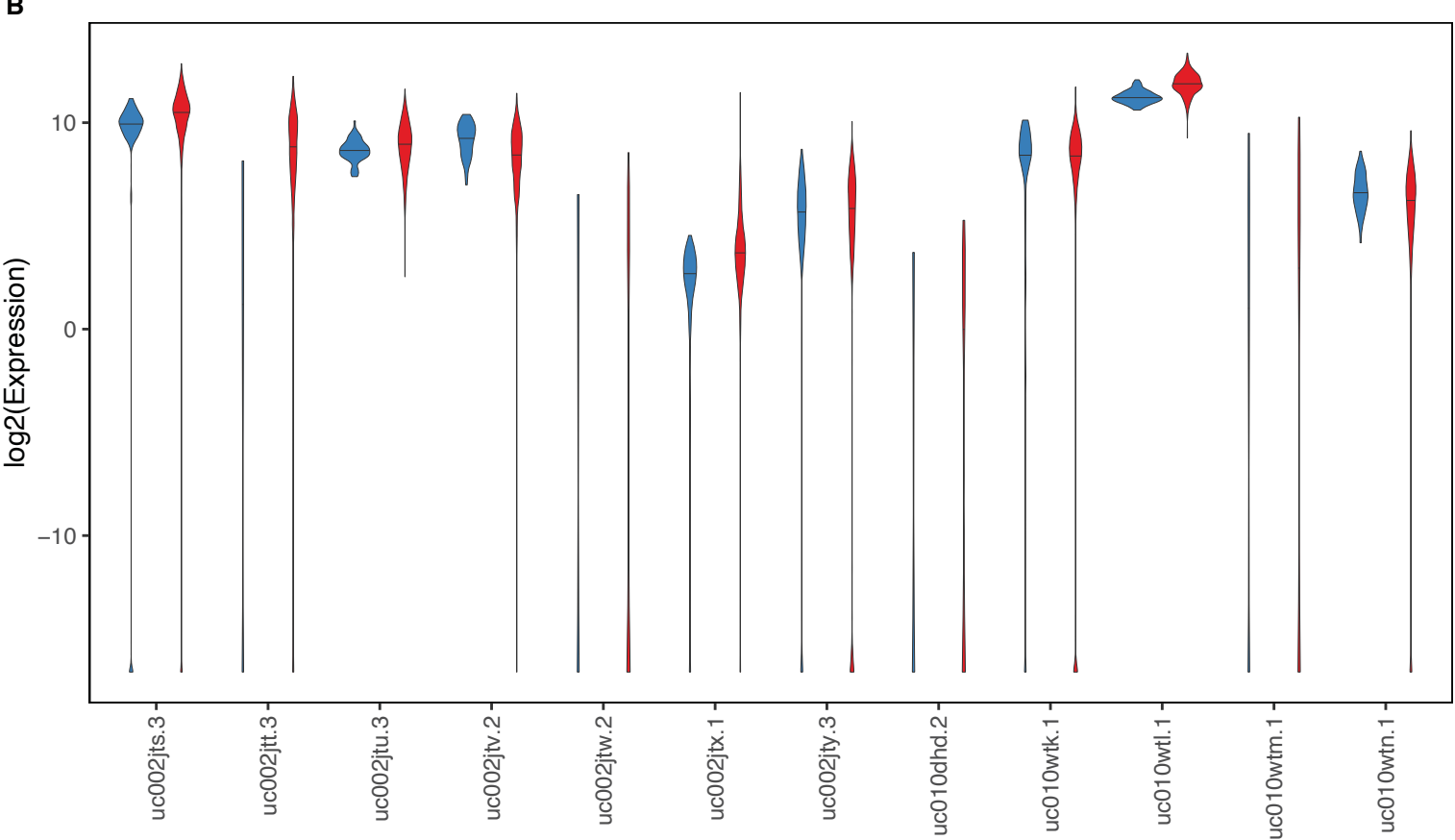

Supplement: Supplementary Figure S13 — SEPT9 isoform promoter methylation and expression in TCGA A. Average SEPT9 isoform promoter methylation levels in TCGA LUAD samples. n.m.d, nonsense mediated decay; processed_tran., processed transcript; r.i., retained intron. A pseudocount of 0.00001 was added to each value. Lines represent median values. B. Average expression of SEPT9 isoforms in TCGA LUAD samples. [file mmc14.pdf]

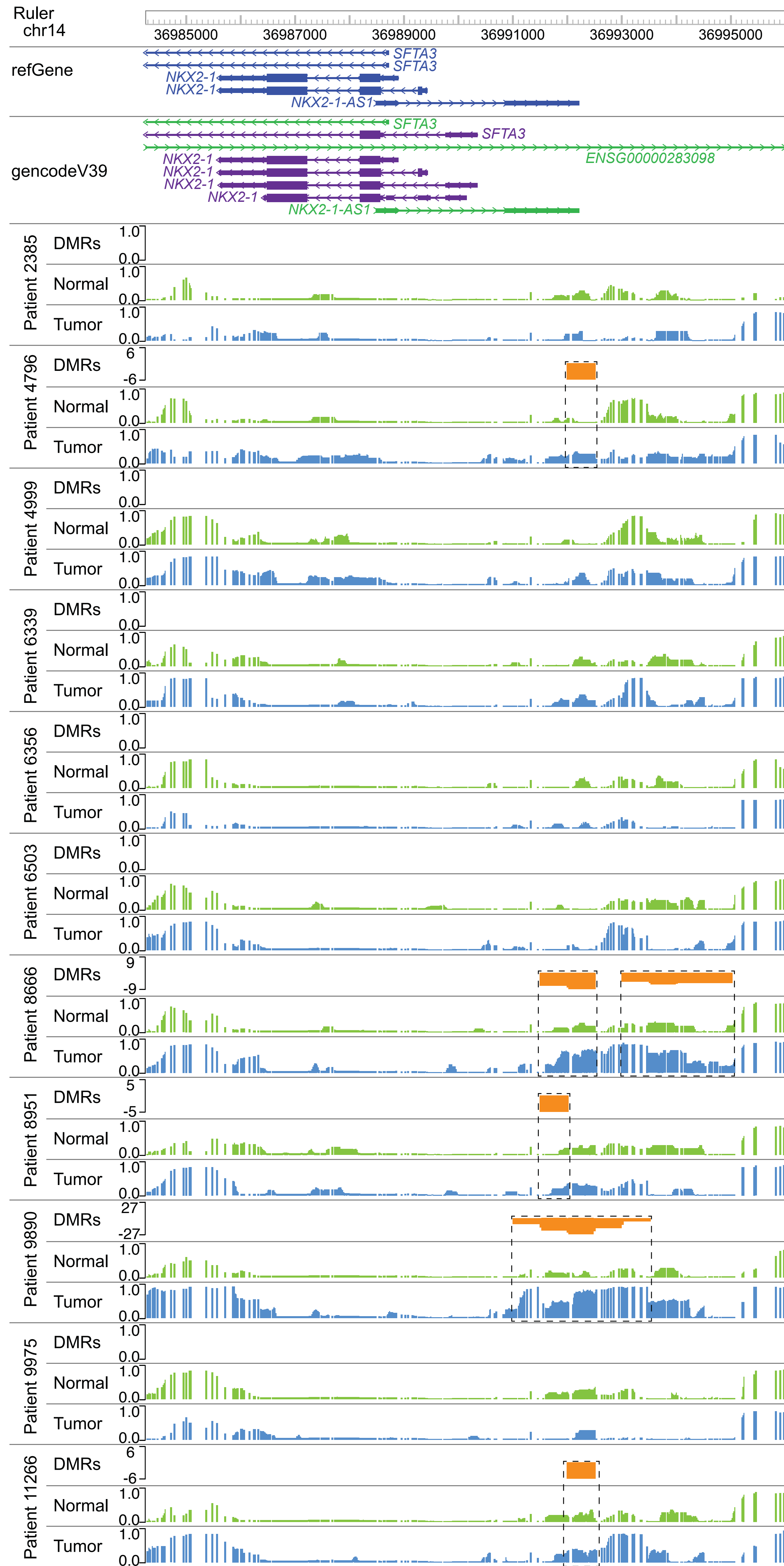

Supplement: Supplementary Figure S14 — Genome browser view of NKX2-1 promoter methylation Genome browser view of the DNA methylation levels over the NKX2-1 promoter for patients with both normal and tumors samples. All samples are grouped by patient, where three tracks are shown per patient: a track highlighting the locations of DMRs (top, orange), the methylation of the corresponding normal sample (middle, green), and the methylation of the corresponding tumor sample (bottom, blue). Methylation values range from 0 (unmethylated) to 1 (methylated), and methylation levels corresponding to DMRs are outlined by dotted lines. [file mmc15.pdf]

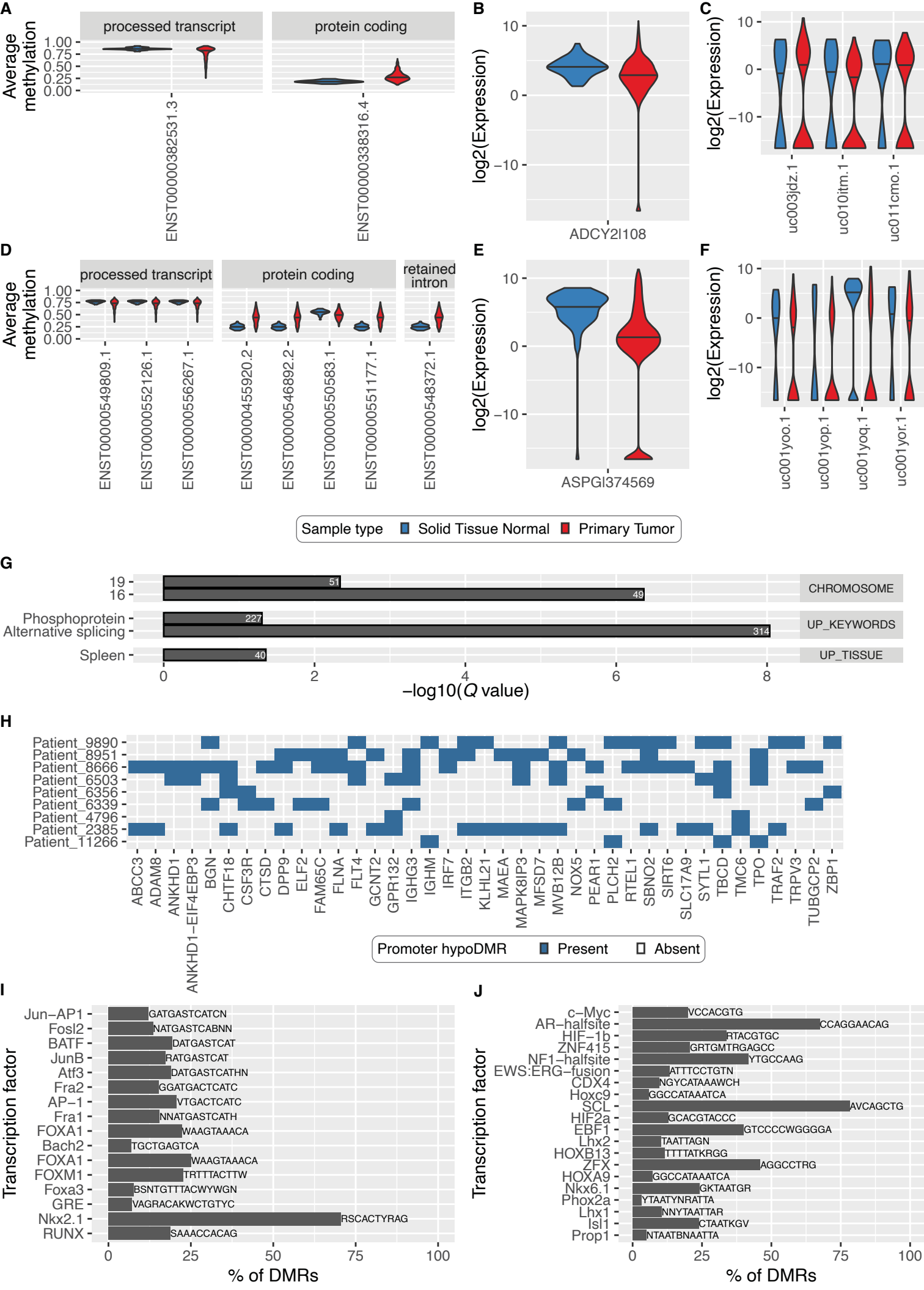

Supplement: Supplementary Figure S15 — Enriched binding motifs and gene pathways for DMRs overlapping with gene promoters A. Average methylation of each ADCY2 transcript promoter in TCGA LUAD samples and matched normal lung. B.ADCY2 expression in TCGA samples. A pseudocount of 0.00001 was added to each value. C.ADCY2 isoform expression level in TCGA samples. A pseudocount of 0.00001 was added to each value. D. Average methylation of each ASPG transcript promoter in TCGA LUAD samples and matched normal lung. E.ASPG expression in TCGA samples. A pseudocount of 0.00001 was added to each value. F.ASPG isoform expression level in TCGA samples. A pseudocount of 0.00001 was added to each value. Lines in panels A–F represent median values. G. Significantly enriched gene sets among genes with a hypoDMR in the promoter in at least two patient comparisons, as determined by DAVID. Only terms with a Benjamini-corrected P < 0.05 are included. Terms are labeled with the number of selected genes and ordered by corrected P within each category. H. Indication of DAVID Spleen UP_TISSUE Pathway genes with a hypoDMR in the promoter in at least 2 patients, stratified by patient. Top 20 most enriched motifs within DMRs overlapping gene promoters in at least two patient comparisons, as determined by HOMER known motif analysis, for (I) hypoDMRs (n = 458) and (J) hyperDMRs (n = 3050, out of 41 terms). Only motifs with a Benjamini-corrected P < 0.05 are included. Motifs are listed to the right of each bar, and the Y-axis lists the corresponding TF. Motifs are ordered by uncorrected P. TF, transcription factor. [file mmc16.pdf]

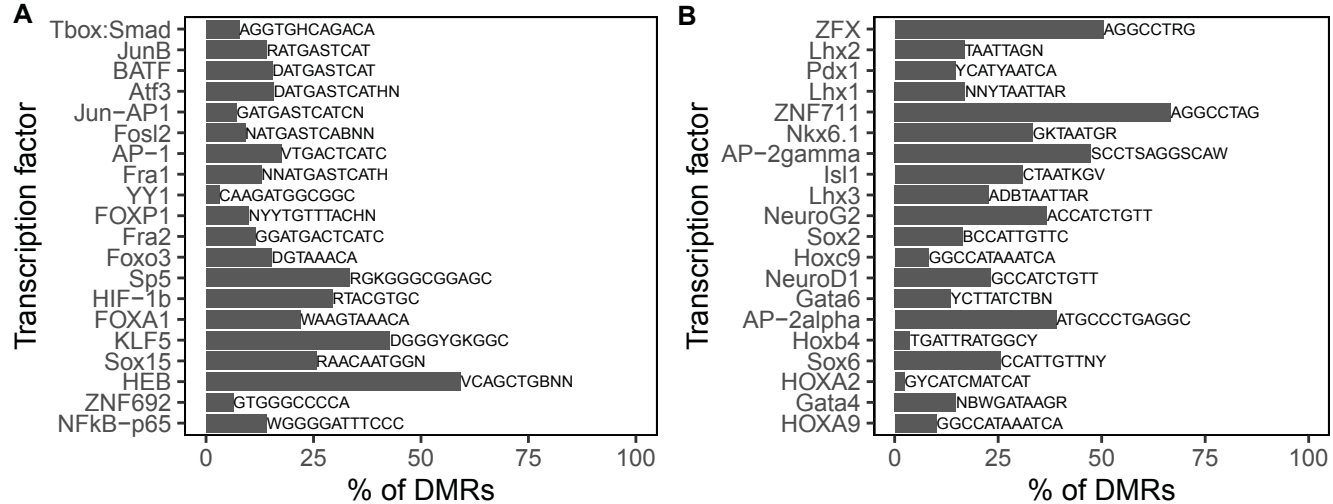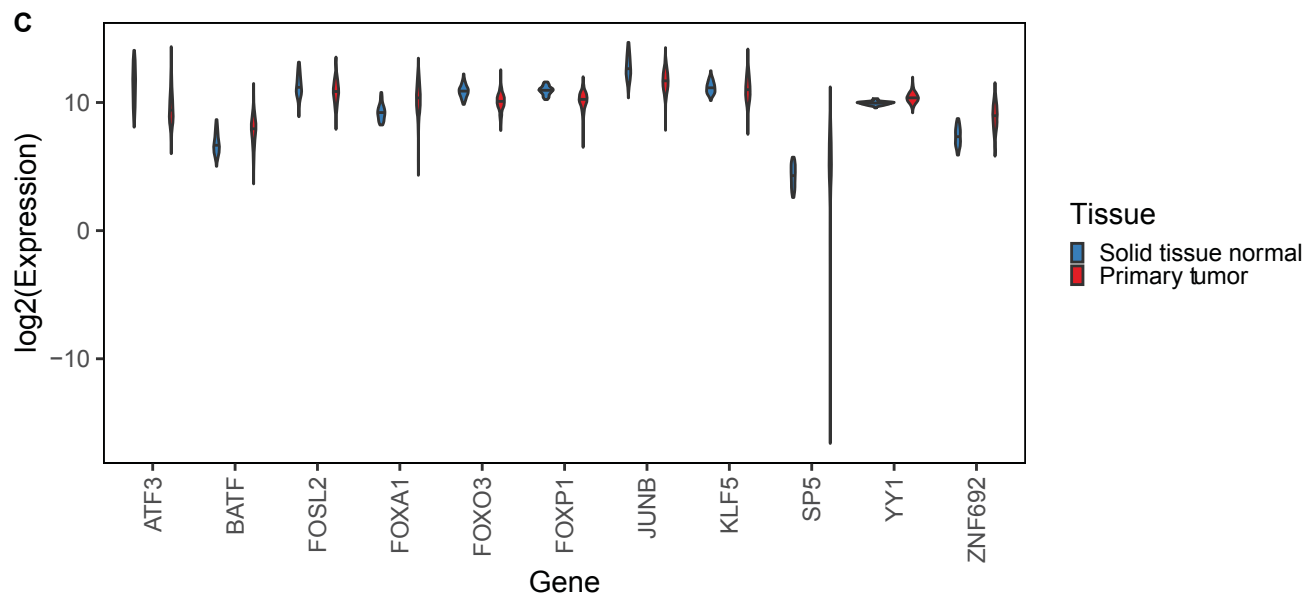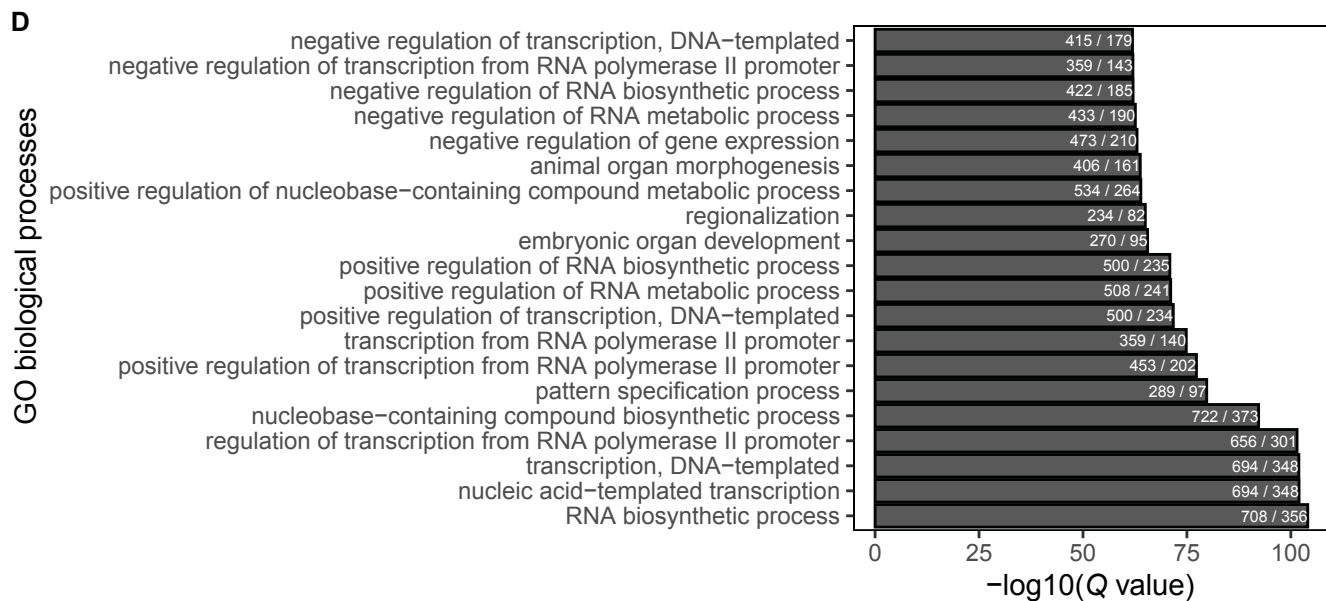

Supplement: Supplementary Figure S16 — Enriched binding motifs and gene pathways for intergenic DMRs Top 20 most enriched motifs within DMRs found in at least two patient comparisons that overlap intergenic regions but not genes or promoters (intergenic-exclusive), as determined by HOMER known motif analysis, for hypoDMRs (n = 1409 DMRs, 22 terms) (A) and hyperDMRs (n = 1365 DMRs, 70 terms) (B). Only motifs with a Benjamini-corrected P < 0.05 are included. Motifs are listed over each bar, and the Y-axis lists the corresponding TF. Motifs are ordered by uncorrected P. C. Expression level in TCGA LUAD samples of TFs with motifs enriched in intergenic hypoDMRs, split by sample type. Violin plot lines indicate median. A pseudocount of 0.00001 was added to avoid undefined values. Wilcox P < 0.001 for all but KLF5. D. Top 20 significant GO biological processes for intergenic-exclusive DMRs that are hypermethylated in at least two patients (out of 394 terms), as identified by GREAT (see Materials and methods). Terms are ordered by FDR-corrected binomial Q and are labeled by the number of DMRs over the number of genes involved. [file mmc17.pdf]

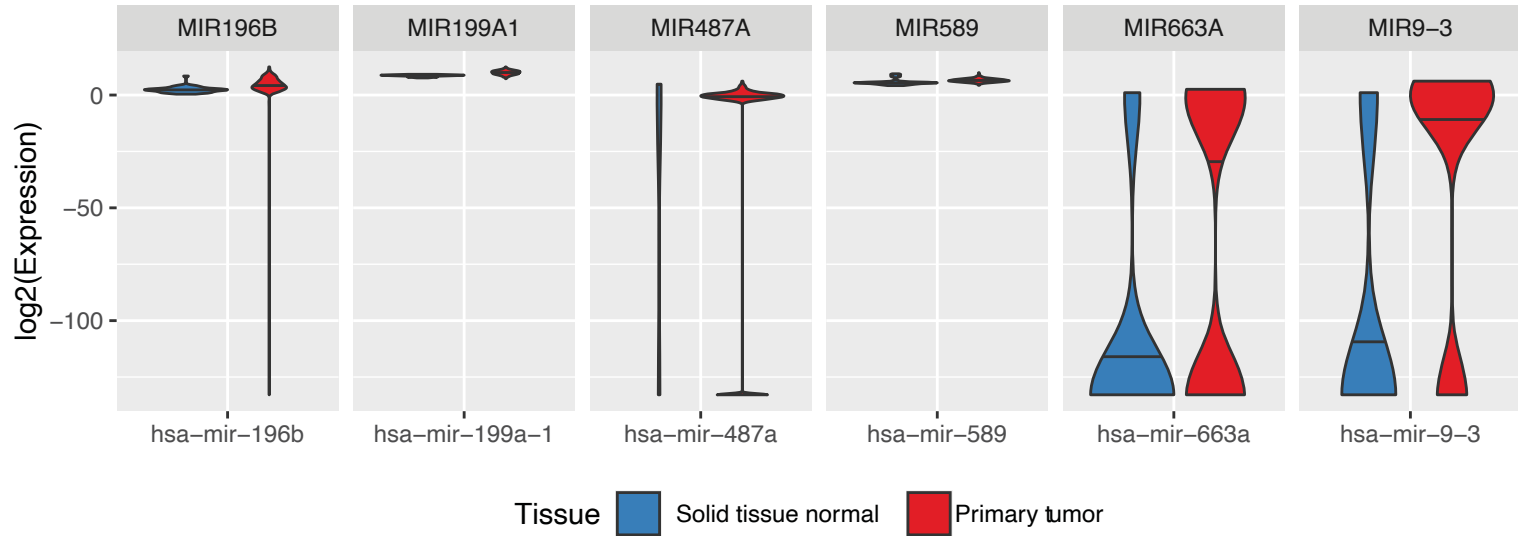

Supplement: Supplementary Figure S17 — TCGA expression level of miRNA overlapping with recurrent DMRs miRNA expression level in TCGA LUAD and normal lung samples for miRNA that overlapped a DMR in multiple patients and had significantly different expression between TCGA sample types (Wilcox P < 0.01). A pseudocount of 1E-40 was added to avoid undefined values. Violin lines indicate median values. The corresponding GENCODE gene name is listed above each graph. See Materials and methods for details on linking miRNA and GENCODE IDs. miRNA, microRNA. [file mmc18.pdf]

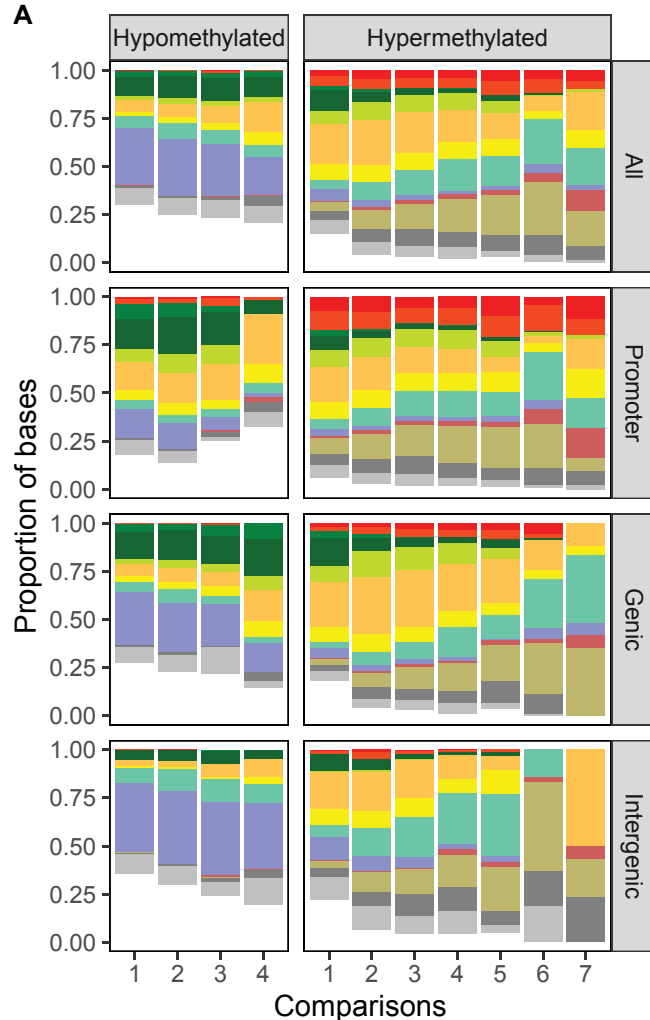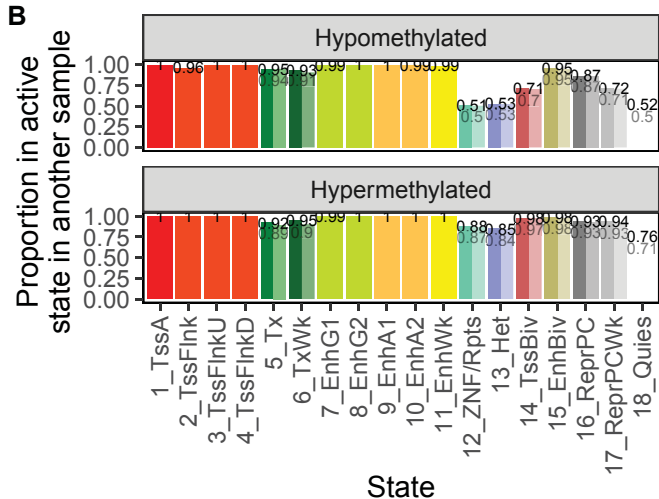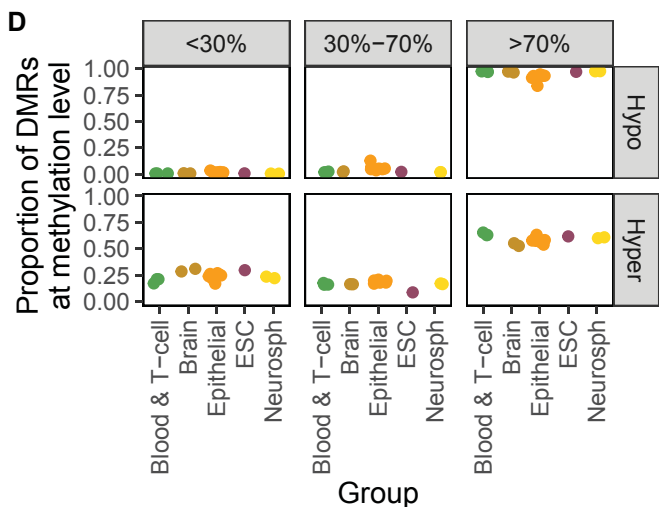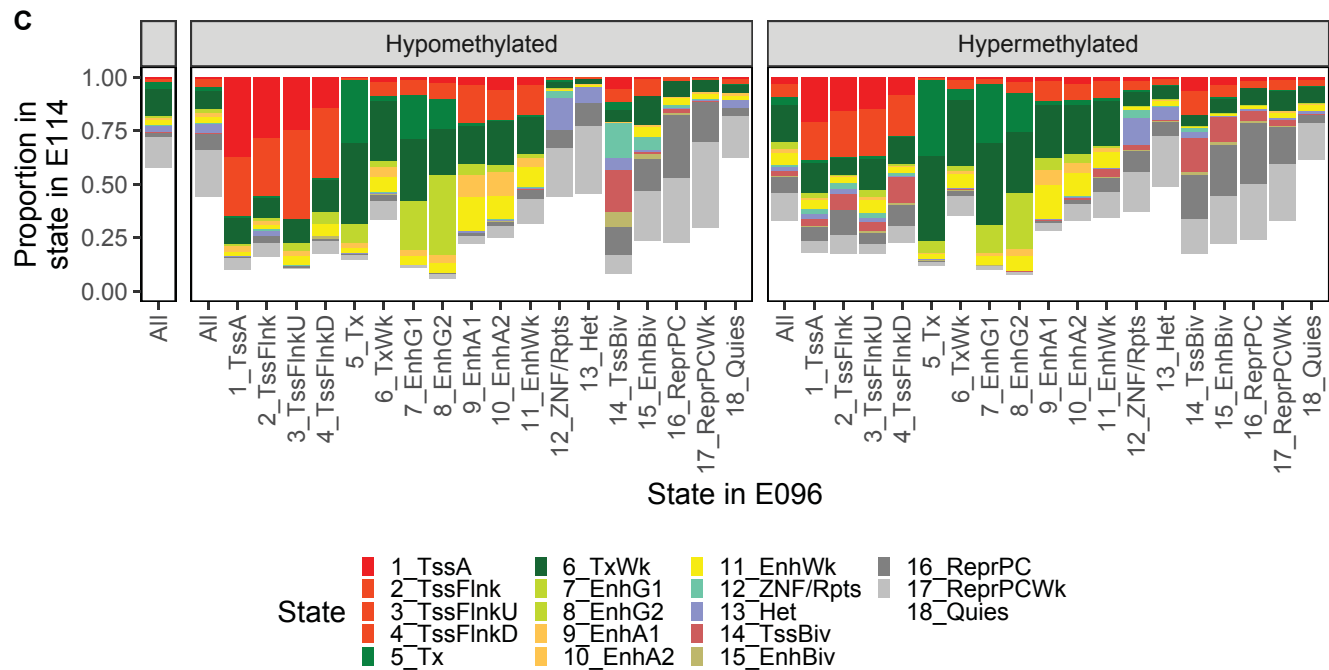

Supplement: Supplementary Figure S18 — Epigenetic state of DMRs in Roadmap tissues A. Proportion of Roadmap sample E096 (adult lung) genomic bases overlapping hypoDMRs or hyperDMRs in each 18-state ChromHMM state, split by exclusive feature overlap and the number of patient comparisons in which the DMR was found (maximum 4 comparisons for hypoDMRs, 7 comparisons for hyperDMRs to avoid categories with < 10 DMRs). B. Proportion of DMRs in an active 15-state ChromHMM state in a Roadmap sample besides E096, split by DMR direction, state in E096 (X-axis), and whether the DMR was in an active state in E096 (True, bold; False, faded). Proportions are listed above each bar. C. Proportion of Roadmap sample E114 (A549 lung carcinoma cell line) bases in each 18-state ChromHMM state, overall or overlapping hypoDMRs or hyperDMRs, and for DMRs split by ChromHMM state in E096. The overall state in E114 was restricted to regions overlapping 500 bp bins that contained CpGs. D. Proportion of DMRs at each average methylation level in each Roadmap sample, split by sample group and DMR direction in the patient-matched NSCLC samples. [file mmc19.pdf]

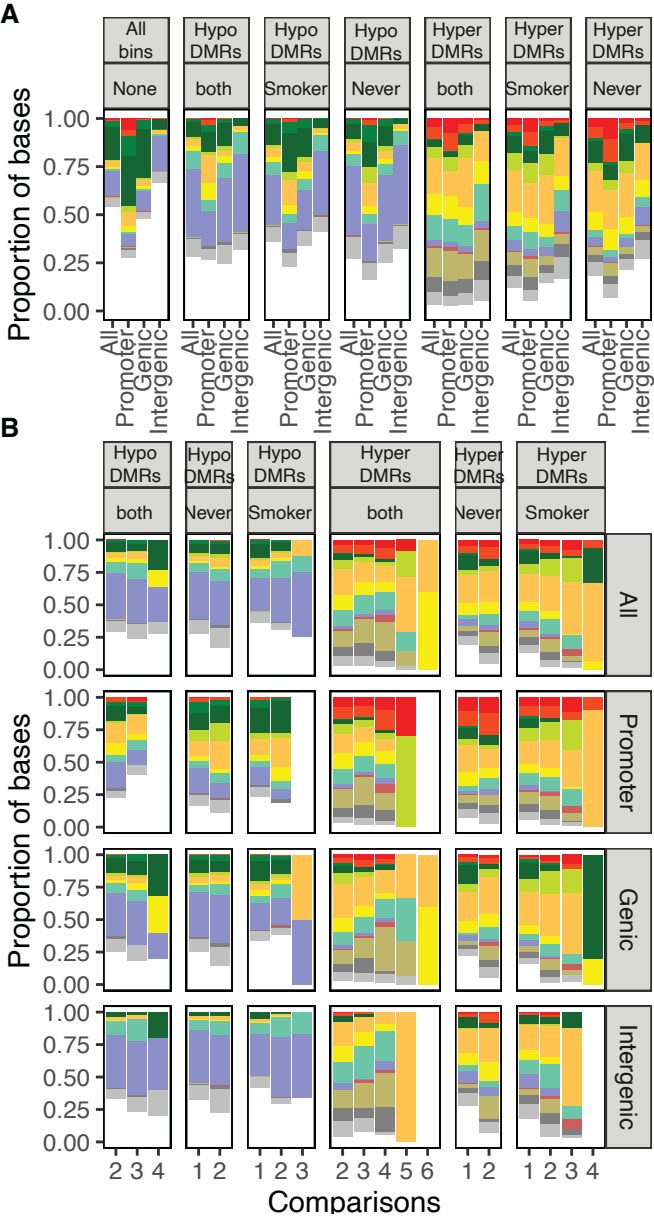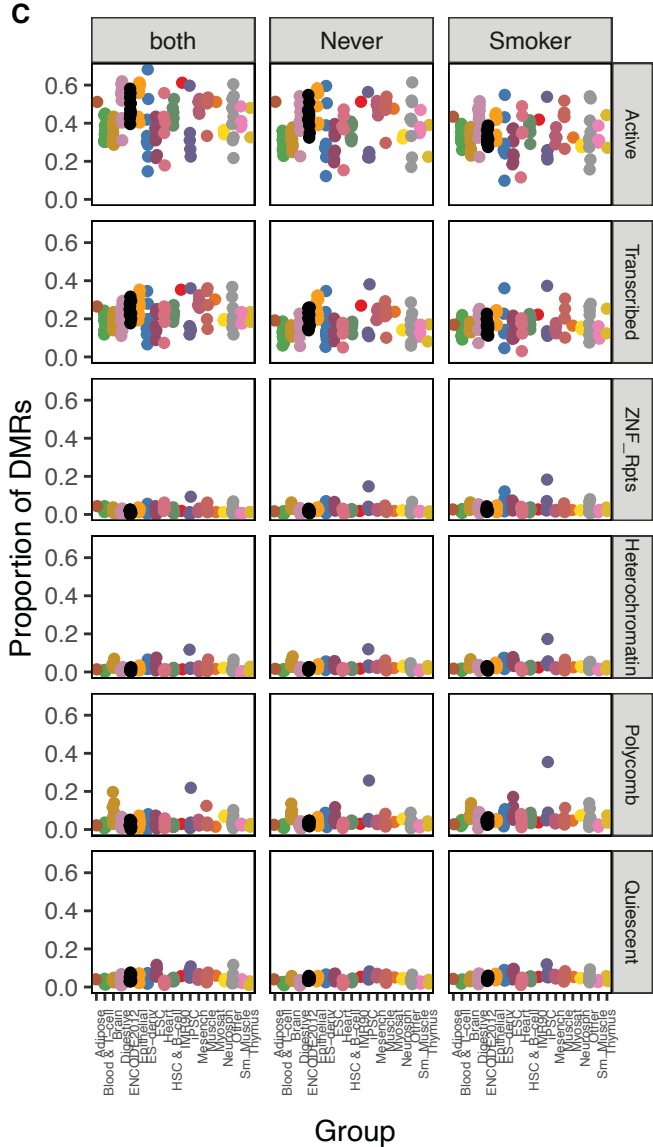

Supplement: Supplementary Figure S19 — Epigenetic state of DMRs in Roadmap tissues, according to smoking status A. Proportion of Roadmap sample E096 (adult lung) bases in each 18-state ChromHMM state, overall and overlapping hypoDMRs or hyperDMRs that were either exclusive to smokers, exclusive to never-smokers, or found in both, split by exclusive feature overlap. Genic DMRs overlapped genes but not promoters, and intergenic DMRs did not overlap genes or promoters. The overall state in E096 was restricted to regions overlapping 500 bp bins that contained CpGs. B. Proportion of Roadmap sample E096 (adult lung) bases overlapping hypoDMRs or hyperDMRs either exclusive to smokers, never-smokers, or found in both, in each 18-state ChromHMM state, split by exclusive feature overlap and the number of patient comparisons in which the DMR was found. C. The proportion of hypoDMRs exclusive to smokers, never-smokers, or found in both, in an active 15-state ChromHMM state in each Roadmap sample, by sample group (columns) and its 18-state ChromHMM state in E096 (rows) (see Materials and methods for composite state definitions). [file mmc20.pdf]

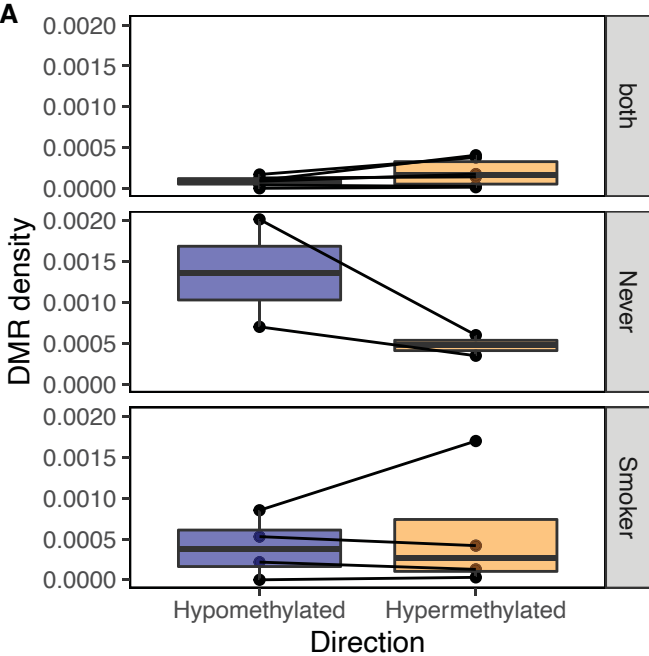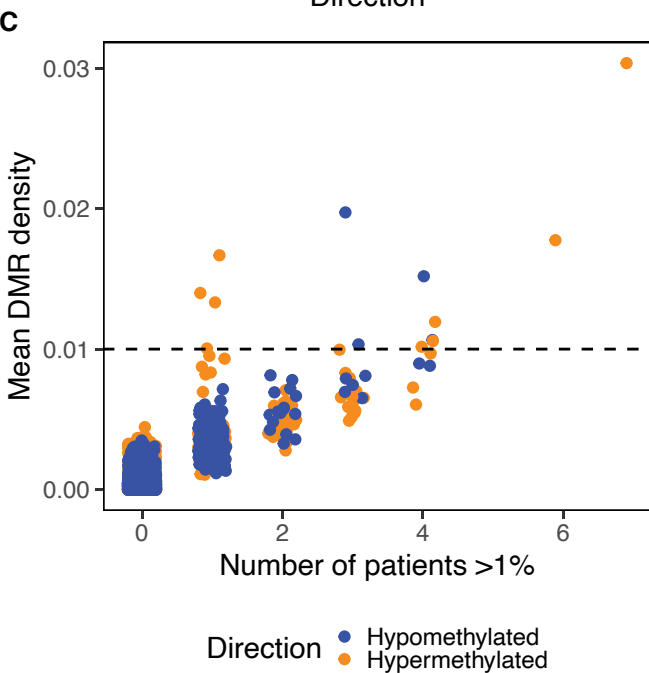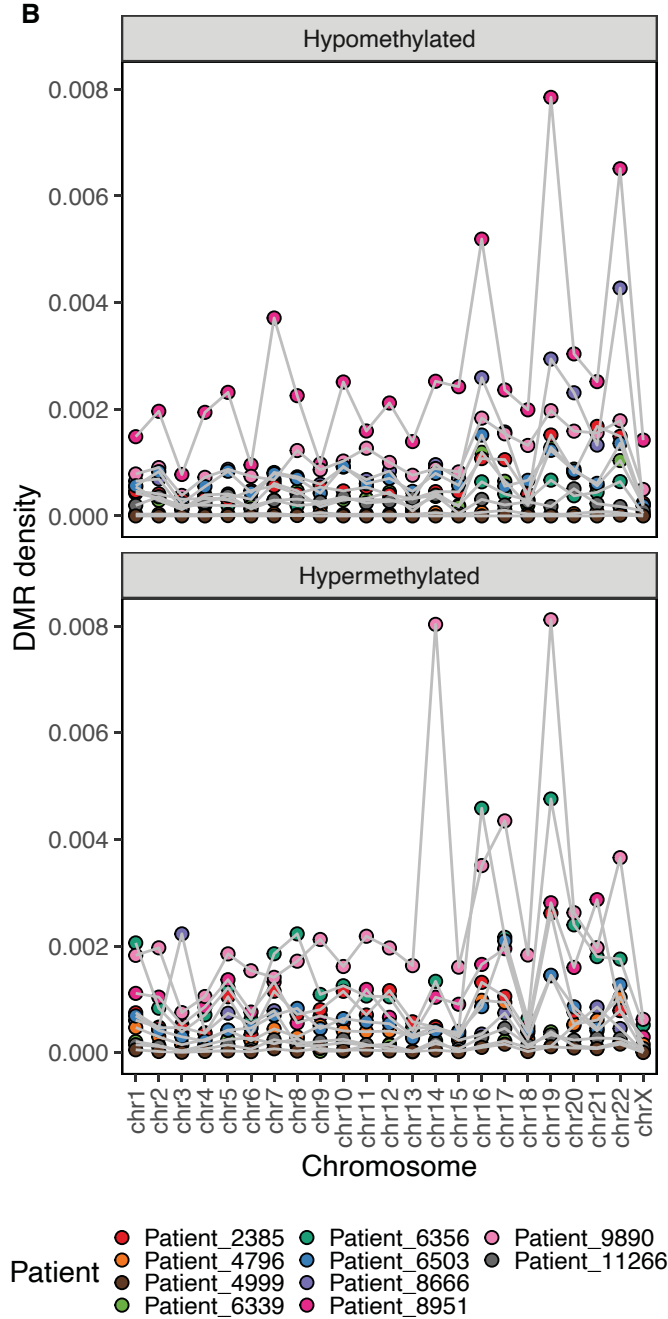

Supplement: Supplementary Figure S20 — DMR density across patients and according to smoking status A. Genome-wide DMR density per patient (number of DMRs versus number of 500 bp bins overlapping CpGs), by DMR direction, for DMRs exclusive to smokers (bottom), never-smokers (middle), or identified in both (top). B. DMR density per patient and chromosome, by DMR direction. C. Mean DMR density of each 1 Mb window across all patients versus the number of patients in which the DMR density was > 1%, by DMR direction. Dashed line indicates a mean DMR density of 1%. Windows that did not overlap a CpG were omitted. [file mmc21.pdf]

Density, DMRs

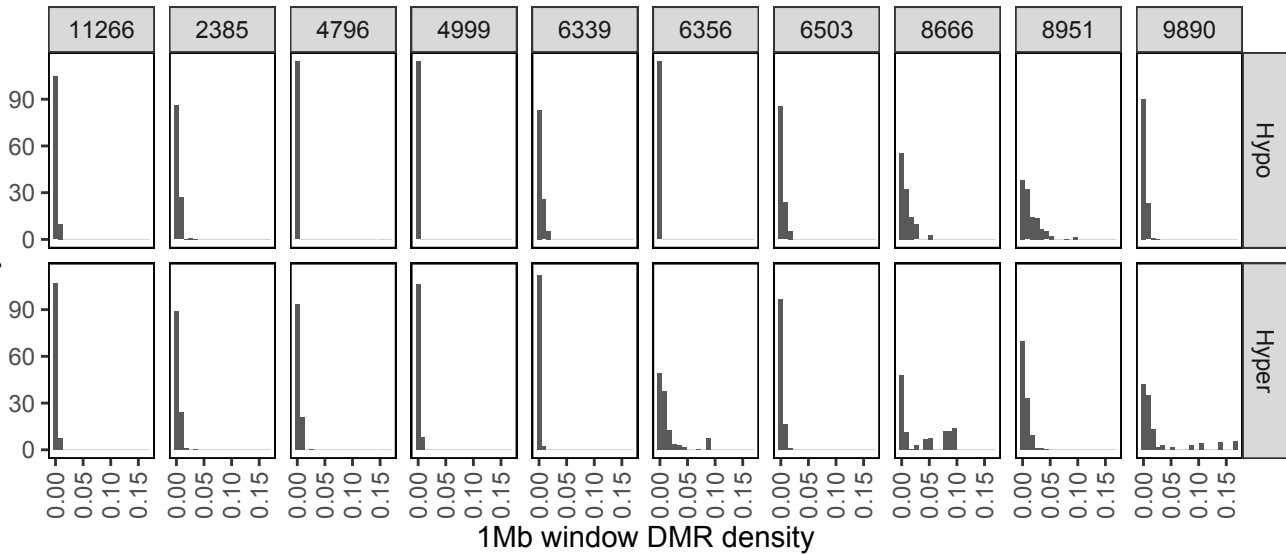

Supplement: Supplementary Figure S21 — Proportion of DMRs in high-density windows Density distribution of the proportion of DMRs exclusive to that patient that were within 1 Mb windows of each DMR density, by patient and DMR direction. [file mmc22.pdf]

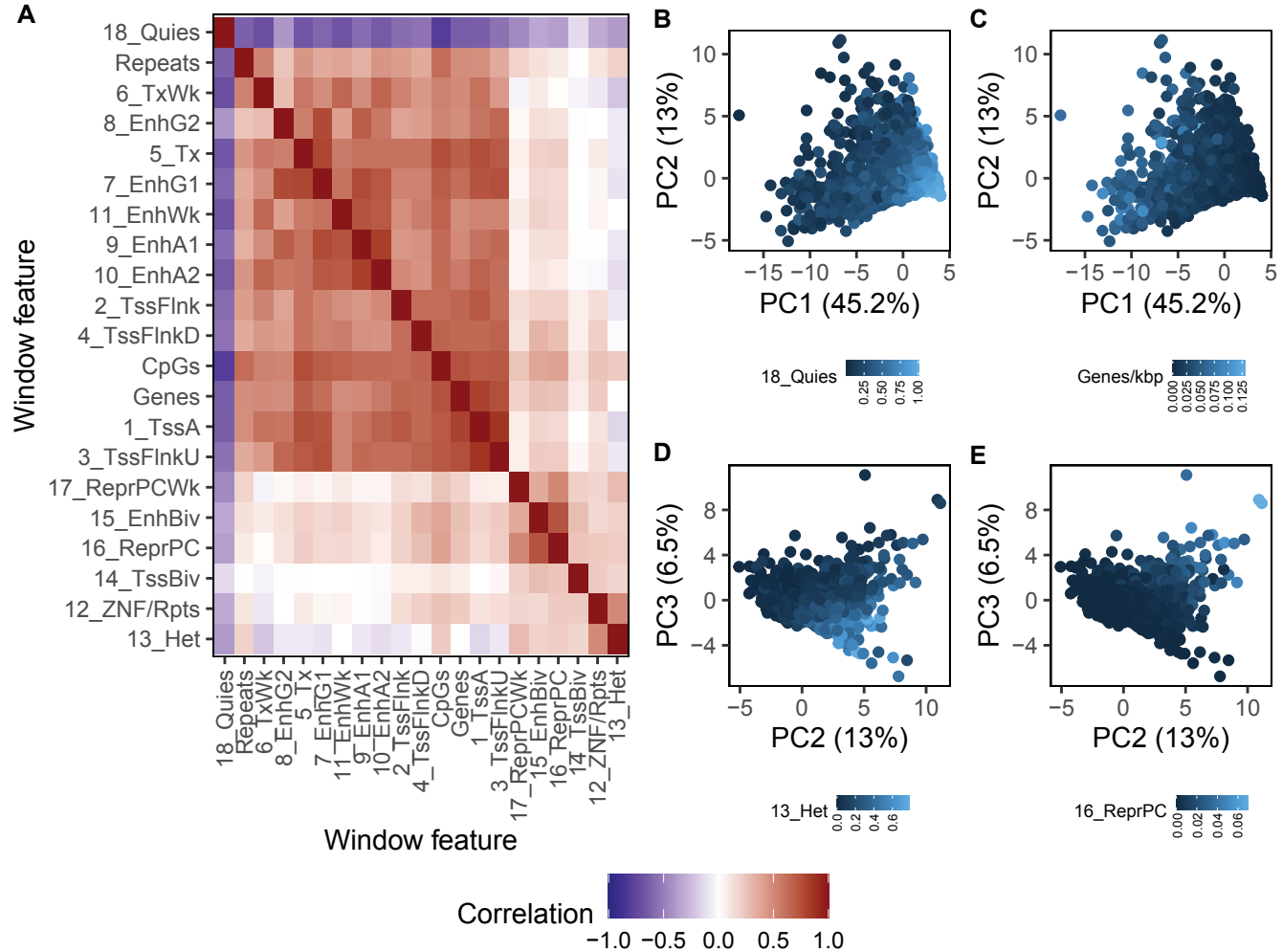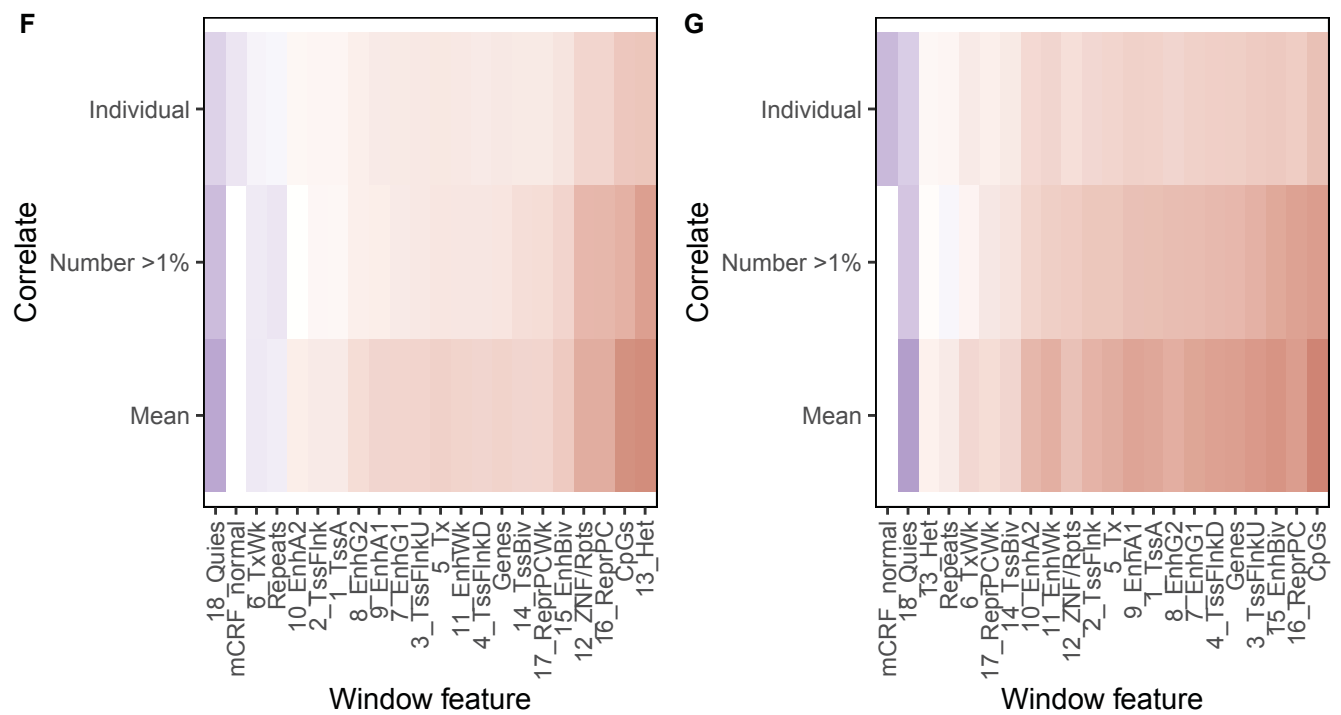

Supplement: Supplementary Figure S22 — Window features correlating with DMR density A. Pearson correlation between 1 Mb window features, including proportion in each 18-state ChromHMM state in E096 and gene, CpG, and repeat density. Features are ordered via unsupervised hierarchical clustering with complete clustering. PCA on 1 Mb windows, using the features described in panel A. Windows are colored by proportion in 18_Quies in E096 (B), gene density (C), proportion in 13_Het in E096 (D), and proportion in 16_ReprPC in E096 (E). Axis titles display the amount of variance explained by each PC. Pearson correlation between DMR density and 1 Mb window features for hypoDMRs (F) and hyperDMRs (G), including proportion in each 18-state ChromHMM state in E096, gene, CpG, and repeat density, and average CpG methylation level in the corresponding normal sample (“mCRF_normal”). Correlations are presented for DMR density in individual comparisons (“Individual”), the number of patients in which the density was > 1% (“Number > 1%”), and the mean DMR density across patients (“Mean”). Features are ordered by median correlation. Missing values are in grey. [file mmc23.pdf]

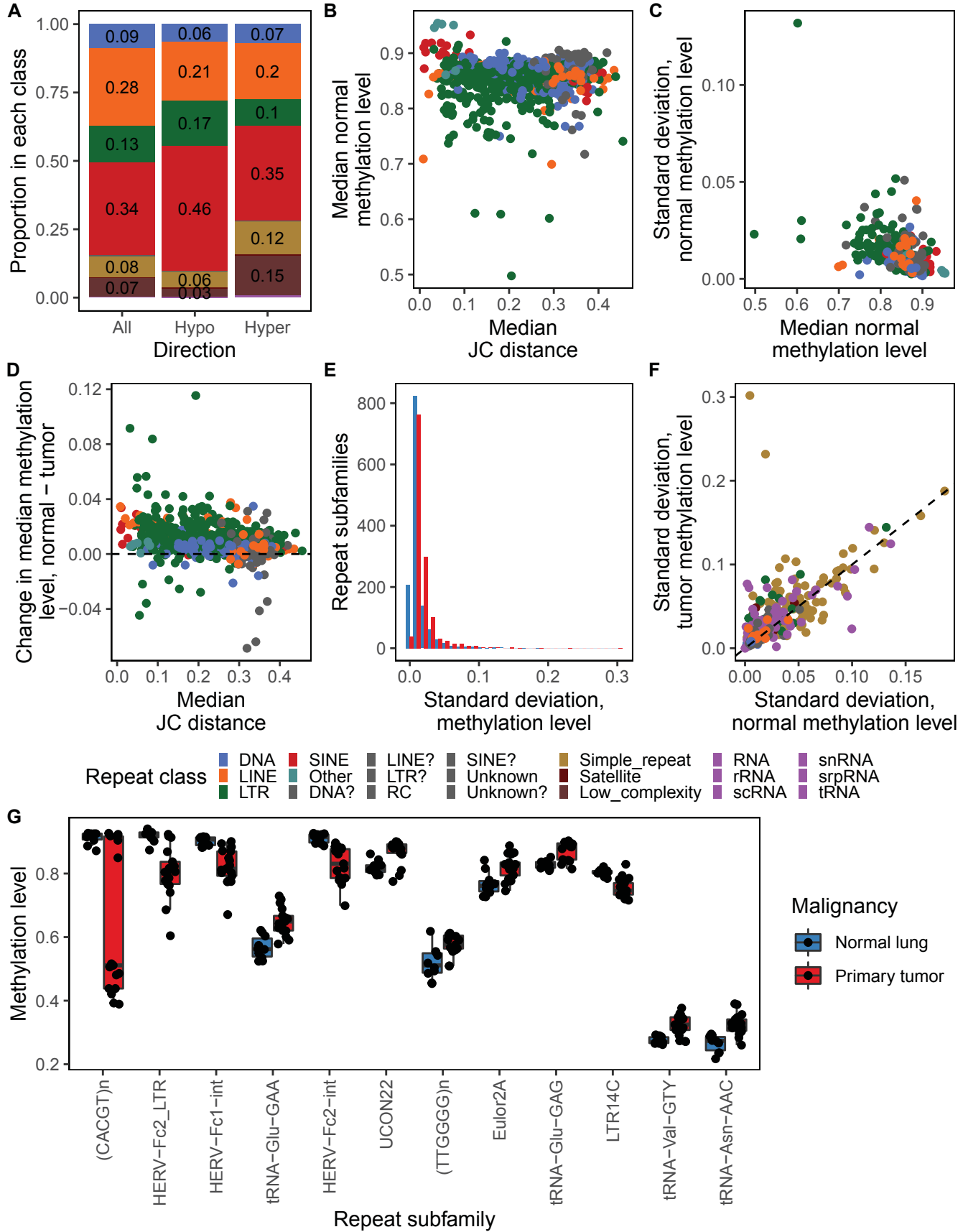

Supplement: Supplementary Figure S23 — Repeat subfamily methylation level alternations in NSCLC A. Proportion of repeats in each class, overall and overlapping any hypoDMR or hyperDMR. Proportions < 0.02 are not shown. B. Median subfamily Jukes–Cantor distance versus the median average CpG methylation level in normal lung samples, TE subfamilies only. C. Median subfamily average CpG methylation level in normal lung samples versus standard deviation across normal lung samples, TE subfamilies only. D. Median subfamily Jukes–Cantor distance versus the change in median average CpG methylation level between normal lung and primary tumor samples, TE subfamilies only. Dashed line indicates no change in median methylation. E. Distribution of standard deviations in subfamily average CpG methylation level across normal lung and tumor samples. F. Standard deviation in subfamily average CpG methylation level in normal lung versus tumor samples. Dashed line indicates identity. G. The average methylation level in all normal lung and tumor samples, for repeat subfamilies with a change in median average methylation between normal lung and tumors samples ≥ 0.05 and a Wilcox P < 0.01. Ordered by absolute change in median average methylation. JC, Jukes–Cantor. [file mmc24.pdf]
