# Supplementary material for: Non-small Cell Lung Cancer Epigenomes Exhibit Altered DNA Methylation in Smokers and Never-smokers
Source: Genomics Proteomics Bioinformatics. 2023 Sep 22;21(5):991–1013. doi: 10.1016/j.gpb.2023.03.006 (PMC10928376; doi:10.1016/j.gpb.2023.03.006)
Supplement: Supplementary Figure S24 — Enrichment of repeat subfamilies for overlap with DMRs A. Log odds ratio enrichment of DMRs over repeat subfamilies, colored by repeat class, by DMR direction. Only subfamilies overlapping > 5 DMRs across all patients are shown. Dashed line represents no enrichment or depletion. B. Subfamily CpG density versus LOR enrichment for overlap with DMRs, by DMR direction, TE subfamilies only. [file mmc25.pdf]

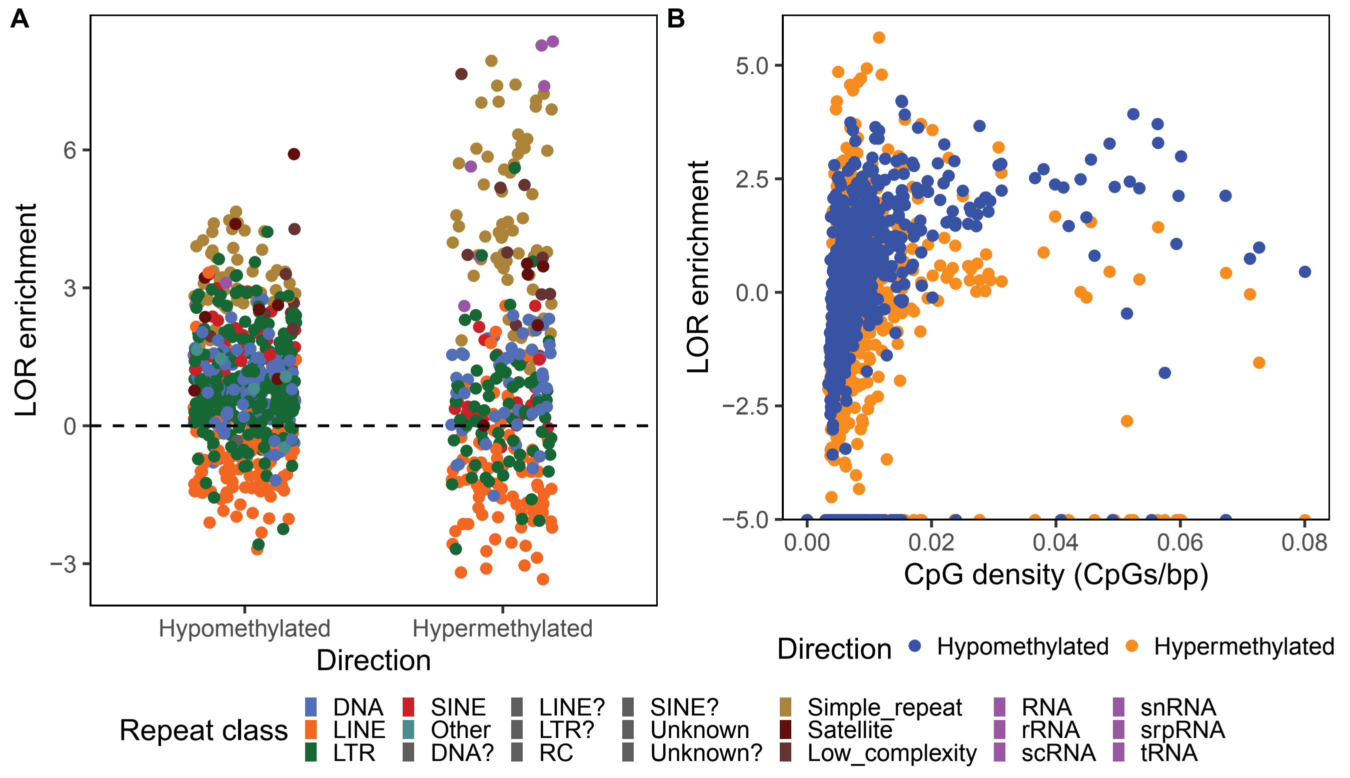
A
B
LOR enrichment
6
3
0
−3
Hypomethylated
Hypermethylated
Direction
5.0
2.5
0.0
−2.5
−5.0
0.00
0.02
0.04
0.06
0.08
CpG density (CpGs/bp)
Direction
Hypomethylated
Hypermethylated
Repeat class
DNA
LINE
LTR
SINE
Other
DNA?
LINE?
LTR?
RC
SINE?
Unknown
Unknown?
Simple_repeat
Satellite
Low_complexity
RNA
rRNA
scRNA
snRNA
srpRNA
tRNA
